# Molecular and in vivo studies of a glutamate-class prolyl-endopeptidase for coeliac disease therapy

**DOI:** 10.1038/s41467-022-32215-1

**Published:** 2022-08-01

**Authors:** Laura del Amo-Maestro, Soraia R. Mendes, Arturo Rodríguez-Banqueri, Laura Garzon-Flores, Marina Girbal, María José Rodríguez-Lagunas, Tibisay Guevara, Àngels Franch, Francisco J. Pérez-Cano, Ulrich Eckhard, F. Xavier Gomis-Rüth

**Affiliations:** 1 Proteolysis Laboratory; Department of Structural Biology, Molecular Biology Institute of Barcelona (CSIC), Barcelona Science Park; c/Baldiri Reixac, 15-21, 08028 Barcelona, Catalonia Spain; 2 Section of Physiology; Department of Biochemistry and Physiology; Faculty of Pharmacy and Food Science, University of Barcelona, Av. Joan XXIII, 27-31, 08028 Barcelona, Catalonia Spain; 3 Research Institute of Nutrition and Food Safety (INSA-UB), University of Barcelona, Av. Prat de la Riba, 171, 08921 Santa Coloma de Gramenet, Catalonia Spain

**Keywords:** Enzyme mechanisms, Molecular medicine, X-ray crystallography, Proteases

## Abstract

The digestion of gluten generates toxic peptides, among which a highly immunogenic proline-rich 33-mer from wheat α-gliadin, that trigger coeliac disease. Neprosin from the pitcher plant is a reported prolyl endopeptidase. Here, we produce recombinant neprosin and its mutants, and find that full-length neprosin is a zymogen, which is self-activated at gastric pH by the release of an all-β pro-domain via a pH-switch mechanism featuring a lysine plug. The catalytic domain is an atypical 7+8-stranded β-sandwich with an extended active-site cleft containing an unprecedented pair of catalytic glutamates. Neprosin efficiently degrades both gliadin and the 33-mer in vitro under gastric conditions and is reversibly inactivated at pH > 5. Moreover, co-administration of gliadin and the neprosin zymogen at the ratio 500:1 reduces the abundance of the 33-mer in the small intestine of mice by up to 90%. Neprosin therefore founds a family of eukaryotic glutamate endopeptidases that fulfils requisites for a therapeutic glutenase.

## Abstract (web summary)

Celiac disease is characterized by intolerance to gluten, a cereal protein. Here, the authors show that neprosin, a glutamate peptidase from the pitcher plant, efficiently cleaves gluten components under physiological conditions in vitro and in the gut of mice.

## Introduction

Coeliac disease (CoD) is a chronic autoimmune enteropathy that affects individuals with genetic and environmental sensitization to dietary gluten, a group of cereal prolamin storage proteins rich in proline and glutamine^1,2^. Prolamins that trigger CoD include gliadin and glutenin in wheat, hordein in barley, and secalin in rye. Intestinal damage can be inflicted by as little as ~10 mg of dietary gluten per day^3^, which is <0.1% of the amount found in a typical western diet^2^. CoD is a global health burden across all age ranges, with a worldwide serological prevalence of 1.4%^4^ that increases by 7.5% every year^5^. The disease is caused by partially degraded gluten peptides, including a 33-residue fragment of wheat α-gliadin (33-mer) that is immunogenically the most relevant^2,6^. These peptides resist further cleavage by gastric, pancreatic and intestinal brush-border membrane peptidases owing to their high proline content (13 in the 33-mer). In coeliacs, they cross the mucosal epithelium of the small intestine, where the glutamine residues are deamidated by tissue transglutaminase. This enhances the affinity of the peptides for the DQ2.5/DQ2.2 and DQ8 alleles of the human leukocyte antigen (HLA) receptor, which are necessary for the development of CoD^2^. Receptor binding triggers a severe pro-inflammatory autoimmune response mediated by T cells, with intestinal effects including intraepithelial lymphocytosis, crypt hyperplasia, atrophy of small-intestine villi and mucosal inflammation^2^. These lead to the chronic malabsorption of nutrients, diarrhoea, vomiting, bloating, abdominal pain and intestinal lymphomas. Extraintestinal manifestations include delayed puberty, osteoporosis, axonal neuropathy and cerebellar ataxia^7^, which reduce the life expectancy of coeliacs. There is no treatment for CoD, so patients must adhere to a lifelong strict gluten-free diet, which restores the normal architecture of the intestinal villi^2^. However, gluten-free diets do not provide balanced nutrition^7^, and many coeliacs suffer intestinal symptoms even with adherence to such dietary restrictions^8,9^. Moreover, gluten is found in most processed foods and medicines, making dietary compliance challenging in western societies^2^. This has created a demand for effective CoD therapies.

One promising approach is the development of endopeptidases that cleave the toxic peptides and would thus act as bona fide glutenases for oral enzyme therapy^10–12^, reminiscent of lactase tablets for lactose intolerance^13^. Such an approach would also benefit patients suffering from non-coeliac gluten sensitivity, which has a worldwide prevalence of up to 13%, and irritable bowel syndrome, with a prevalence of <0.5%^8,14,15^. A candidate glutenase must fulfil certain criteria for clinical application. First, it should work in the stomach during digestion, before the gastric bolus passes into the duodenum and initiates the autoimmune response, and thus must remain stable and active in the acidic gastric environment (pH ~2.5) as well as resisting gastric pepsin. Second, a reasonable dose should efficiently digest gliadin and the 33-mer when combined with pepsin under gastric conditions, which requires the processing of large quantities of dietary protein. Third, it should not harm intestinal structures or inhibit nutrient absorption, and thus ideally should be inactive at the slightly acidic postprandial pH of the duodenum^16^.

The therapeutic potential of several glutaminyl and prolyl endopeptidases (PEPs) has been assessed, representing various catalytic classes and diverse sources including bacteria, fungi, insects and germinating cereals^7,10–12^. These include a serine PEP from *Aspergillus niger*^10,17^; STAN1, a combination of *A. niger* aspartate aspergillopepsin and *Aspergillus oryzae* serine dipeptidyl-peptidase IV^18^; latiglutenase, a combination of a glutamine-specific cysteine peptidase from barley and a modified serine prolyl-specific oligopeptidase from *Sphingomonas capsulata*^19^; subtilisin-type serine endopeptidases from the natural oral colonizers *Rothia aeria* and *Rothia mucilaginosa*^11^; and the synthetic enzymes KumaMax and Kuma062/TAK-062, developed by the computational redesign of kumamolysin, a serine endopeptidase from the bacterium *Alicyclobacillus sendaiensis*^20^. However, none of these candidates fulfils all of the above requirements. The current frontrunners do not show high activity under gastric pH conditions and/or require very high doses or protective modifications, such as PEGylation or microencapsulation. Accordingly, clinical trials have not yet achieved significant clinical remission in coeliacs and have not demonstrated the ability of these enzymes to replace a gluten-free diet^12^. Worse, many so-called enzyme preparations currently sold over the counter as CoD dietary supplements do not inactivate toxic gluten peptides and thus represent a hazard for coeliacs^21^.

Neprosin is a 380-residue endopeptidase of unknown class and mechanism, currently assigned to family U74 in the MEROPS database (www.ebi.ac.uk/merops^22^). It is a PEP that was discovered in the digestive fluid of the carnivorous plant *Nepenthes* × *ventrata*, which traps prey animals in its pitcher^23–26^. The enzyme might have a function in protein metabolism during prey digestion and/or defence^23^. In combination with other peptidases from the digestive fluid, it has been identified as part of a potential glutenase preparation^24^. Purified neprosin is also considered a useful reagent for proteomics^25,26^.

Here, we establish a human recombinant production system to produce high yields of neprosin. We determine its mechanism of activation in vitro as well as its thermal stability, pH profile, general proteolytic and peptidolytic activities, and susceptibility to a panel of peptidase inhibitors. We also test cleavage of gliadin and the 33-mer in vitro to evaluate the ability of neprosin to act as a solo glutenase. Moreover, we evaluate the effect of recombinant neprosin on the processing of gliadin in mice. Finally, we report the crystal structure of the neprosin zymogen and its mature form in product-mimicking complexes. These data reveal the mechanism of latency, the overall and active-site architectures, catalytic mechanism and peptidase class, which have been validated by a cohort of mutants.

## Results and Discussion

### Heterologous expression, autolytic maturation and stability analysis

**Fig. 1:**
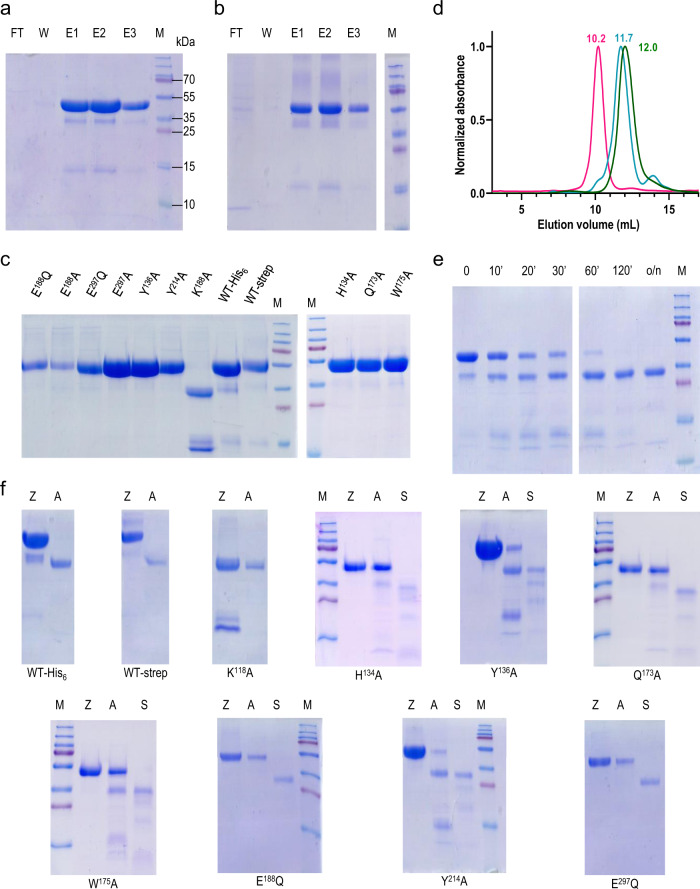
Protein purification and activation. **a** Purification of wild-type (WT) pro-neprosin by His_6_- or (**b**) Strep-tag affinity chromatography. The flow-through (FT), wash (W) and elution (E1–E3) fractions were analysed by SDS-PAGE and Coomassie staining, alongside molecular mass markers (lane M). Panels are representative of three independent experiments. **c** Pro-neprosin mutants (K^118^A, H^134^A, Y^136^A, Q^173^A, W^175^A, E^188^A, E^188^Q, Y^214^A, E^297^Q and E^297^A) after His_6_-tag affinity purification compared with the WT forms of (**a**) and (**b**). Figure representative of two independent experiments. **d** Size exclusion chromatography profiles of pro-neprosin with His_6_ tag (magenta), neprosin with Strep tag (green) and neprosin with His_6_ tag (blue) separated on a Superdex 75 10/300 GL column. Each curve is labelled with the elution volume in mL, representing monomers in all cases. **e** Autolytic maturation of pro-neprosin over time at 37 °C in an acidic buffer. Figure representative of two independent experiments. **f** Activation of pro-neprosin variants (Z lanes) by acidic autolysis (A lanes) or in trans by adding Strep-tagged neprosin (S lanes). Mutant K^118^A (third panel) was obtained as a pre-activated protein after affinity purification, revealing separate PD and mature protein bands (lane Z), which became fully activated by incubation in an acidic buffer (lane A). Figure representative of two independent experiments. For the distinct panels of this figure, relevant source data are provided as a Source Data file if adequate.

Previous studies of neprosin mainly used the enzyme purified from pitcher plant fluid because heterologous expression in *Escherichia coli* produced only a partially impure enzyme with a modest yield^24,25^. We were unable to reproduce this approach so we developed a system based on human cells, assuming that eukaryotic post-translational processing is required. This yielded ~10 mg/L of pure well-folded full-length protein with a C-terminal hexahistidine (His_6_) tag (41 kDa) or ~8 mg/L with a twin-streptavidin (Strep) tag (43 kDa) (Fig. 1a, b). The protein was properly folded and remained stable for several weeks at 4 °C in a neutral buffer, but lacked proteolytic activity, which we attributed to the full-length protein being the pro-neprosin zymogen. Indeed, it readily underwent autolytic maturation at bond P^128^–S^129^ (residue numbering of neprosin in superscript; UniProt ID C0HLV2) over time when incubated in a highly acidic buffer, yielding the neprosin catalytic domain (CD) and the excised pro-domain (PD) (Fig. 1e). The latter was eventually degraded, and both pro-neprosin and neprosin migrated as monomers when checked by calibrated size-exclusion chromatography (SEC) (Fig. 1d).

**Fig. 2:**
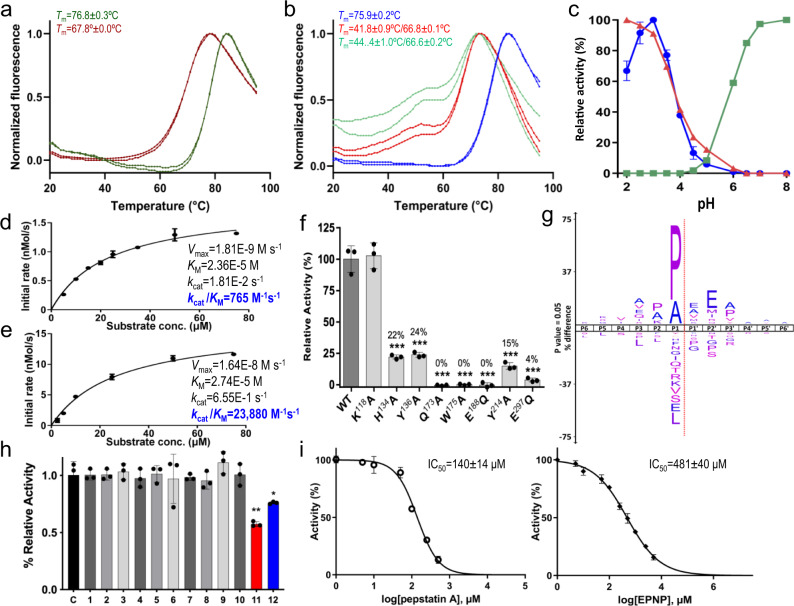
Thermal stability, peptidolytic activity and inhibition assays. **a** Differential scanning fluorimetry showing duplicate curves of temperature-dependent fluorescence variation during the thermal denaturation of neprosin (dark red) and pro-neprosin (green). The inset midtransition temperatures (*T*_m_) are the average inflection points of the two respective curves. **b** Same as (**a**), illustrating the effect of TCEP as a reducing agent at 5 mM (red) and 10 mM (green) compared with untreated pro-neprosin (blue). **c** The pH-dependent activity of pepsin (red), trypsin (green) and neprosin (blue) on a fluorescent BSA substrate. For neprosin, data are means ± SD (*n* = 3 independent experiments). Values for trypsin and pepsin were measured once. **d**, **e** Kinetics of the neprosin-mediated cleavage of the fluorogenic peptides (**d**) FS6 (100 nM neprosin) and (**e**) FS6-QPQL (25 nM neprosin). The insets show the corresponding *V*_max_, *k*_cat_, *K*_M_ and *k*_cat_/*K*_M_ values. **f** Peptidolytic activity of wild-type (WT) neprosin and mutants on the fluorogenic FS6-QPQL peptide. Statistical significance determined by two-sided Student’s *t*-test (*p < 0.1; **p < 0.05; ***p < 0.001). For the seven bars with ***, the *p* values were, left to right, 0.0052, 0.0050, 0.0036, 0.0036, 0.0029, 0.0033 and 0.0034. **g** Logo depicting the substrate preference of neprosin based on reanalysis of deposited data^25^. **h** Effect of the test molecules or mixtures (1) 1,10-phenathroline, (2) AEBSF, (3) phosphoramidon, (4) marimastat, (5) cOmplete, (6) BGP, (7) captopril, (8) DAN, (9) BEOPC, (10) AMP, (11) pepstatin A and (12) EPNP compared to the WT control (C). Only the last two compounds achieve significant inhibition. Statistical significance determined by two-sided Student’s *t*-test (*p < 0.1; **p < 0.05; ***p < 0.001). For the two bars with ** and *, the *p* values were 0.0215 and 0.0698, respectively. **i** Plot of the inhibitory activity of pepstatin A (left) and EPNP (right) showing tester concentrations with the derived IC_50_ values. For panels **d**–**f**, **h** and **i**, data are means ± SD (*n* = 3 independent experiments) and relevant source data are provided as a Source Data file if adequate.

Differential scanning fluorimetry using the thermofluor approach^27^ revealed a midtransition temperature (*T*_m_) of 68 °C for the mature enzyme (Fig. 2a), which is remarkable for a peptidase that works in an ambient temperature range and is more reminiscent of hyper-thermophilic enzymes^28^. Furthermore, the *T*_m_ of the zymogen was 9 °C higher (Fig. 2a), suggesting the PD promotes stability and, possibly, the correct folding of the full-length protein as reported for other zymogens^29^. This was supported by our inability to express mature neprosin (without the PD) using the same expression system. Finally, thermofluor studies in the presence of a reducing agent revealed an unfolding process with two transitions, the first occurring at 42–44 °C (Fig. 2b). This indicated the existence of disulfide bonds that stabilize the protein, as discussed in more detail below.

### Proteolytic activity

We investigated the effect of pH on the cleavage of fluorescent bovine serum albumin (BSA) by neprosin, using gastric pepsin, an aspartate peptidase, and pancreatic trypsin, a serine peptidase, for comparison (Fig. 2c). The pH optimum of neprosin was 3, close to that of gastric pepsin (pH < 2). By contrast, the pH optimum of trypsin was 8, a value at which both neprosin and pepsin were completely inactive. Pepsin was irreversibly inhibited at neutral pH, as previously reported^30^, whereas neprosin was reversibly activated and inactivated by switching between pH 2.5 and 9.0. Moreover, neprosin was unaffected by freezing or lyophilization at pH 7.5 for storage, thus recovering its full activity after thawing or resuspension in an acidic buffer, respectively. Finally, neprosin was insensitive to cleavage by pepsin at acidic pH. This profile of activity, efficiency, stability and robustness was therefore consistent with a digestive enzyme that must work over prolonged timescales under varying conditions, precisely the natural environment in the pitchers of carnivorous plants^31^.

To gain further insight into the substrate specificity of neprosin and to guide our cleavage assays, we reanalysed published proteomics data based mainly on purified material, which had identified the enzyme as a bona fide PEP^25^. We found 3001 unique cleavage sites spanning **P**_**6**_–**P**_**6**_**′** (substrate and active-site subsite nomenclature based on^32,33^), 1863 (62%) of which featured a proline residue in **P**_**1**_ (Fig. 2g). Proline was also enriched twofold over its natural abundance at **P**_**2**_ and **P**_**3**_**′**, but was strongly disfavoured at **P**_**1**_**′** and **P**_**2**_**′**. Glutamate and methionine were enriched threefold at **P**_**2**_**′**, alanine was readily accepted throughout **P**_**6**_–**P**_**6**_**′**, and glycine was significantly disfavoured at **P**_**1**_–**P**_**3**_**′**. These data revealed a strong preference for substrates with proline at **P**_**1**_ and that specific positions within **P**_**6**_–**P**_**6**_**′** were unsuited for certain amino acids (Fig. 2g).

### Cleavage of gliadin and the 33-mer

**Fig. 3:**
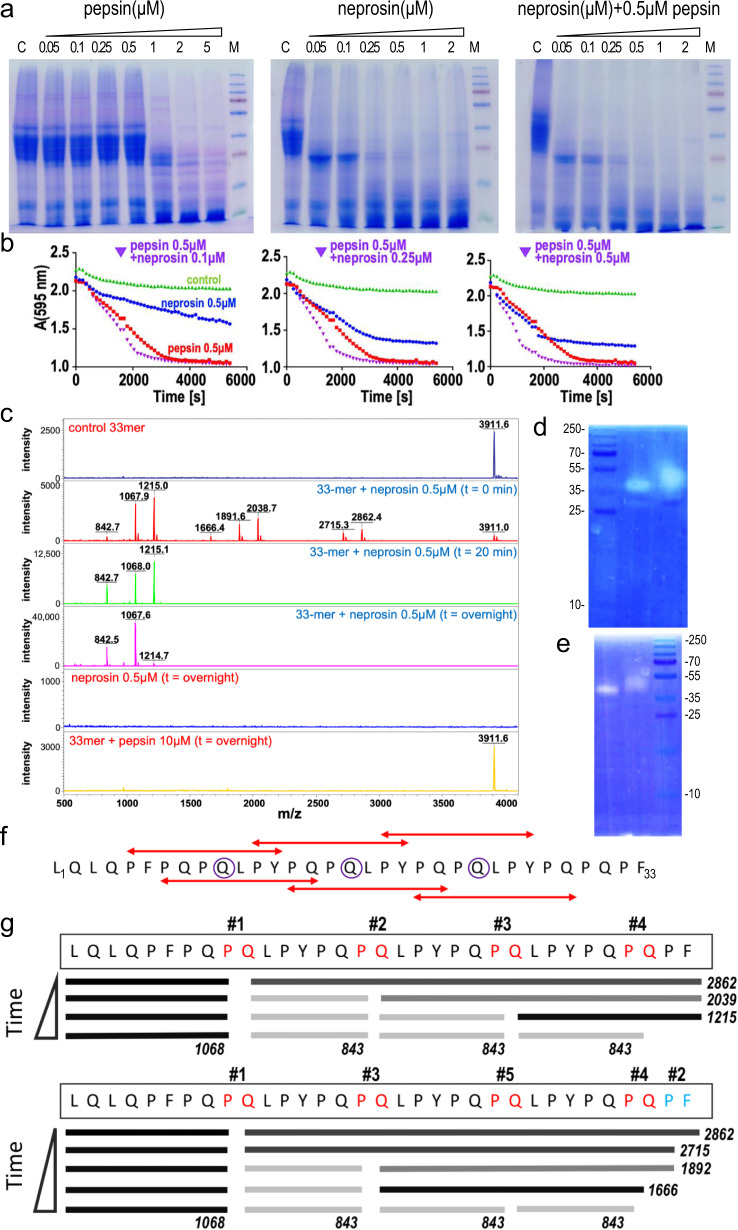
Neprosin activity against molecules relevant for coeliac disease. **a** SDS-PAGE analysis of gliadin exposed to increasing concentrations of pepsin (left), neprosin (centre) or neprosin plus pepsin (right). Figure representative of two independent experiments. **b** Curves depicting gliadin cleavage as in (**a**) over time measured by turbidimetry. **c** Mass spectra of, top to bottom, the 33-mer peptide (3912 Da); the 33-mer peptide after incubation with 0.5 μM neprosin for 0 min, 20 min and overnight; neprosin alone; and the 33-mer peptide after overnight incubation with 10 μM pepsin, which leaves the peptide intact. **d** Gliadin zymogram depicting the activity of neprosin (left lane) and the mature enzyme resulting from pro-neprosin self-activation (right lane). **e** Same as (**d**) but showing gelatin zymography. **f** Sequence of the 33-mer and extent of the six overlapping HLA-DQ2.5-binding epitopes as highlighted by red double arrows^7^. Glutamines susceptible to deamidation by transglutaminase are shown in purple circles^11^. The peptide corresponds to segment L^76^–F^108^ of α-gliadin (UniProt ID P18573). **g** Cleavage of the 33-mer peptide by neprosin over time proceeds according to two pathways (top and bottom). For the distinct panels of this figure, relevant source data are provided as a Source Data file if adequate.

We investigated the ability of neprosin to digest gliadin in the presence and absence of pepsin by SDS-PAGE and turbidimetry, compared to pepsin alone (Fig. 3a, b). Both enzymes efficiently degraded gliadin separately at concentrations below ~5 μM, the physiological threshold of pepsin^34^, but optimal results were achieved when both enzymes were combined. Remarkably, the optimal concentration of neprosin was similar to that of gastric pepsin, and orders of magnitude lower than that required for current glutenase candidates. Zymography showed that neprosin degraded gliadin and gelatine, also a dietary protein, with similar efficiency (Fig. 3d, e).

Next, we investigated cleavage of the 33-mer, which includes three glutamine residues that are deamidated by transglutaminase and six overlapping immunogenic HLA-DQ2.5 T-cell epitopes^6,11,35^, by mass spectrometry (Fig. 3f). We found that peptide at 250 μM was efficiently degraded by 0.5 μM neprosin, a 500:1 ratio, after 20 min at pH 3 (Fig. 3c). No autolytic cleavage products were detected even after overnight incubation, which confirmed the stability of the mature enzyme under acidic conditions. By contrast, pepsin failed to cleave the peptide even after overnight incubation at a 20-fold higher concentration than neprosin, which confirmed the resistance of the 33-mer against digestive peptidases. Analysis of the peptide cleavage fragments generated by neprosin revealed two final products: Q–L–P–Y–P–Q–P (843 Da) and L–Q–L–Q–P–F–P–Q–P (1068 Da). By monitoring the reaction over time (Fig. 3g), we found that cleavage only occurred immediately downstream of five specific proline residues among the 13 present in the 33-mer, preferably at P–Q–P*Q–L–P and always with P–Q–P at the **P**_**1**_–**P**_**3**_ subsites, which qualifies the simple specificity for proline at **P**_**1**_ deduced from indiscriminate proteomics and is in line with disfavouring large hydrophobic residues (leucine, phenylalanine and tyrosine) in **P**_**2**_ as discussed above^25^. Overall, our results demonstrate that the 33-mer is degraded at multiple sites featuring the Q–P*Q–L motif. Remarkably, two P–Q dipeptides are also found in BSA, together with five equally favoured P–E sites (see above), which explains why albumin is a suitable substrate for neprosin at low pH.

Finally, we tested the cleavage of a cohort of fluorogenic peptides. We found that peptide FS6 containing a P–L bond (Mca–K–P–L–G–L–Dpa–A–R–NH_2_), which is a substrate of matrix metalloproteinases and adamalysins^36^, was cleaved with modest efficiency according to kinetic analysis (*k*_cat_/*K*_M_ = 765 M^−1^s^−1^; Fig. 2d). In contrast, peptide variant FS6-QPQL, redesigned to include the neprosin cleavage site of the 33-mer (Mca–Q–P–Q–L–Dpa–A–R–NH_2_), was cleaved 30-fold more efficiently, mainly due to *k*_cat_ increase (*k*_cat_/*K*_M_ = 23,880 M^−1^s^−1^; Fig. 2e). Accordingly, neprosin can be defined as a PEP with a more constrained specificity than P–X that efficiently degrades the 33-mer under gastric-like conditions.

### Inhibitory profile

Given the unknown catalytic class of the enzyme, we next tested a panel of peptidase inhibitors for their ability to block FS6-QPQL cleavage by neprosin (Fig. 2h). We also followed an approach recently applied to find inhibitors of pyrroline-5-carboxylate reductase^37^, whose product is proline, and tested a series of proline-containing/mimicking compounds. We found that only pepstatin A and 2-[(4-nitrophenoxy)methyl]oxirane (EPNP) weakly but significantly inhibited neprosin (Fig. 2h), with half-maximal inhibitory concentration (IC_50_) values of 140 and 480 μM, respectively (Fig. 2i). Given that pepstatin and EPNP-like epoxides are inhibitors of pepsin-type aspartate endopeptidases^38,39^, which share no sequence similarity with neprosin, this pointed to an unexpected peptidase type and mechanism of catalysis for neprosin.

### Evaluation of neprosin activity in vivo

**Fig. 4:**
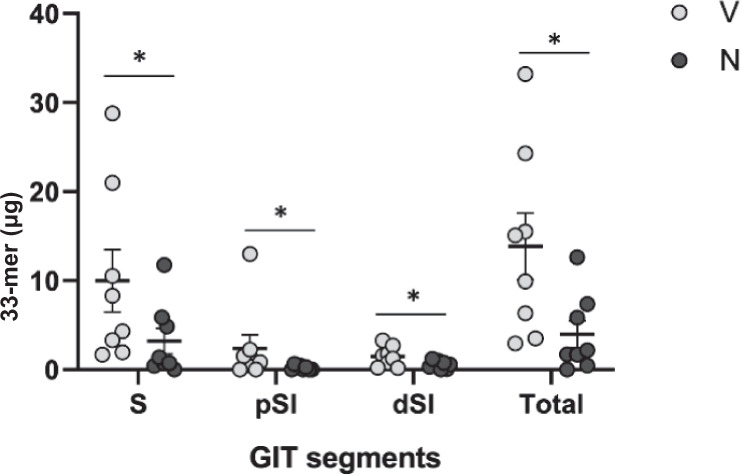
Analysis of neprosin activity against gliadin in vivo. Amount of 33-mer (μg) in the total contents of the stomach (S), proximal small intestine (pSI) and distal small intestine (dSI) of mice receiving neprosin zymogen (N) or vehicle (V) prior to a bolus of gliadin. Results are means ± SEM (*n* = 8 animals per group). Statistical significance was determined by a one-sided F-test (*p < 0.05, N *vs* B). The *p* values were 0.048 (S), 0.049 (pSI), 0.026 (dSI) and 0.021 (Total). Relevant source data are provided as a Source Data file.

To investigate the activity of neprosin in vivo, mice were fed a bolus of gliadin 5 min after receiving either the zymogen at a very low mass ratio (1:500 w/w) or vehicle. After 2.5 h, we harvested the contents of three upper gastrointestinal tract segments and measured the concentration of the 33-mer by enzyme-linked immunosorbent assay (Fig. 4). The peptide was substantially less abundant in all segments of the treated animals (61-91%) and by 71% overall. The inactive zymogen is therefore activated upon reaching the stomach and efficiently helps to break down gliadin (and particularly the 33-mer) in vivo while remaining resistant to physiological digestive enzymes. This occurs at much lower concentrations than those of candidate glutenases, and without protective strategies such as PEGylation or microencapsulation. These results are consistent with a previous study reporting that sensitized NOD/DQ8 mice showed a significant decrease in inflammatory markers when fed gliadin that was pre-digested with pepsin and *Nepenthes* pitcher fluid, which included among other components neprosin and nepenthesin^24^.

### Structural analysis of latent and mature neprosin

**Fig. 5:**
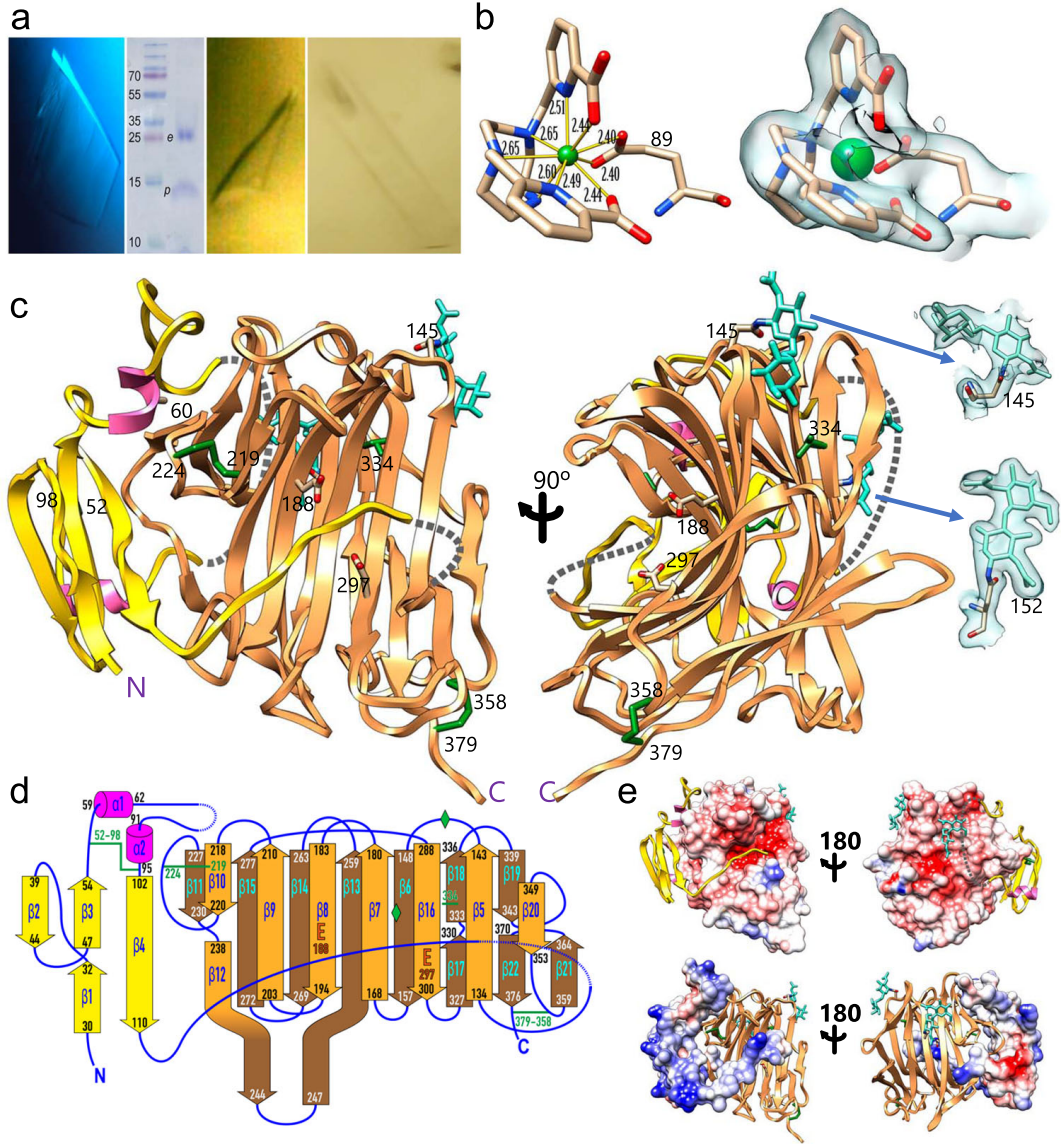
Structures of pro-neprosin and neprosin. **a** Orthorhombic crystals of pro-neprosin (left panel) contained a complex of the cleaved PD (*p*) and the CD (*e*) (centre-left panel). Mature enzyme crystals were monoclinic (centre-right panel, crystal form I; right panel, crystal form II). The experiment of the centre-left panel was performed once. **b** The structure of pro-neprosin was solved using a lutetium derivative. At one site (left panel), the Lu^3+^ cation (green sphere) was nona-coordinated by two carboxylate oxygens plus five nitrogen atoms from the organic scaffold and the carboxylate oxygens of protein residue E^89^ at distances spanning 2.40–2.65 Å. Final (2m*F*_obs_-*DF*_calc_)-type Fourier map of the derivative contoured at 1.3 σ (right panel). **c** Ribbon-type plot of pro-neprosin in frontal (left panel) and lateral (right panel) perspectives. The PD is gold with magenta helices. The mature enzyme is shown in salmon. Disordered/cleaved segments are indicated by grey dashed lines. The two glycosylation sites at N^145^ and N^152^, the seven cysteines, A^60^, and the two catalytic glutamates (E^188^ and E^297^) are shown for their side chains and labelled. The final Fourier map around the two glycan chains is pictured at 0.6 σ. **d** Topology of pro-neprosin with strands as arrows (labelled β1–β22) and the two short helices (α1 and α2) as magenta rods. The terminal residues of each secondary structure element are indicated. The PD has yellow strands and magenta helices, the front sheet of the mature enzyme moiety is in orange, and the back sheet is in brown. The seven cysteines are further indicated in green, the glycans are shown as green rhombi. The catalytic glutamates are marked for reference. **e** The top row shows the front view of pro-neprosin as in (**c**) (left) and the back view (right), both depicting the PD as yellow ribbon and the Coulombic surface of the CD (*red*, –10 kcal/mol·*e*; *blue*, +10 kcal/mol·*e*) computed with *Chimera*^85^. The calculated pI of the mature enzyme component is 4.3. The bottom row shows the same except that here the PD is shown for its Coulombic surface (pI = 9.5) and the CD as salmon ribbon.

We crystallized pro-neprosin in an orthorhombic space group (Fig. 5a and Supplementary Table 1) and found that the polypeptide was cleaved at the physiological maturation site (P^128^–S^129^). The crystals therefore contained the zymogenic complex of the cleaved PD and the CD (Fig. 5a). We solved the structure by single-wavelength anomalous diffraction, collecting data at the lutetium L_III_ absorption edge wavelength from a crystal soaked in Lu-Xo4^40^ (Fig. 5b). This soaking led to significant variation in one of the crystal cell axes when compared to native crystals while keeping good diffraction of X-rays (Supplementary Table 1). The final refined model of the derivative complex was used to solve the native pro-neprosin structure by molecular replacement. Moreover, mature neprosin produced two different monoclinic crystal forms, I and II (Fig. 5a and Supplementary Table 1), whose structures were likewise solved by molecular replacement.

Pro-neprosin is a compact oblong molecule of ~55 × ~45 × ~40 Å (Fig. 5c). The N-terminal PD (R^25^–P^128^) is defined in the final Fourier map from A^29^ onwards, and features a globular part (A^29^–G^112^) followed by a linker (L^113^–P^128^) to the downstream CD (S^129^–Q^380^). Segment (N^122^–N^131^), which includes the cleaved maturation site, is flexible. The PD features an antiparallel three-stranded β-sheet in which the central strand is bisected by the insertion of the leftmost strand (Fig. 5c, d). The two right strands are connected by a long segment on the top, which includes two short α-helices, a disulfide bond (C^52^–C^98^), and a disordered 10-residue segment (Y^77^–N^86^) at the back of the molecule. The latter probably results from the protruding glycan chain attached to N^152^ within a back strand of the CD (Fig. 5c). A second glycan is attached to N^145^ from a cross-over loop on top of the CD. Beyond the last strand of the PD, the chain undergoes a 90° turn and enters the PD/CD linker, which runs in extended conformation along the front surface of the CD.

Atypically for peptidases, which are generally α/β-proteins^41^, the CD is an antiparallel β-sandwich, with a seven-stranded strongly-curled front sheet and an eight-stranded back sheet, which provides a scaffold for the former (Fig. 5c, d). Both sheets are interconnected by nine cross-over loops, including long hairpin β12β13, and two further disulfide bonds (C^219^–C^224^ and C^358^–C^379^) on either side of the sandwich (Fig. 5d). All these elements contribute to a compact and sturdy structure, which explains the remarkable pH stability of neprosin and its ability to resist pepsin digestion. By contrast, the disulfide bonds are not deeply buried in the structure, which explains its sensitivity to reducing agents. The structure of mature neprosin crystal form I (Supplementary Table 1) proved practically identical to the equivalent part of the zymogen, with a core root mean square deviation (RMSD) of 0.62 Å. The only significant difference was encountered at N^232^–Y^233^, which is folded outward in the zymogen to accommodate I^103^ at the beginning of the rightmost strand of the PD. Crystal form II, in turn, was practically indistinguishable from crystal form I (core RMSD = 0.66 Å) except for the tip of loop Lβ21β22, which was spaced apart by 3.8 Å, and the C-terminal tag, which was reoriented owing to crystal packing. Thus, the mature enzyme component is essentially preformed in the zymogen as seen in most peptidases, with the notable exception of chymotrypsin-type serine peptidases^42–44^.

### The active site

**Fig. 6:**
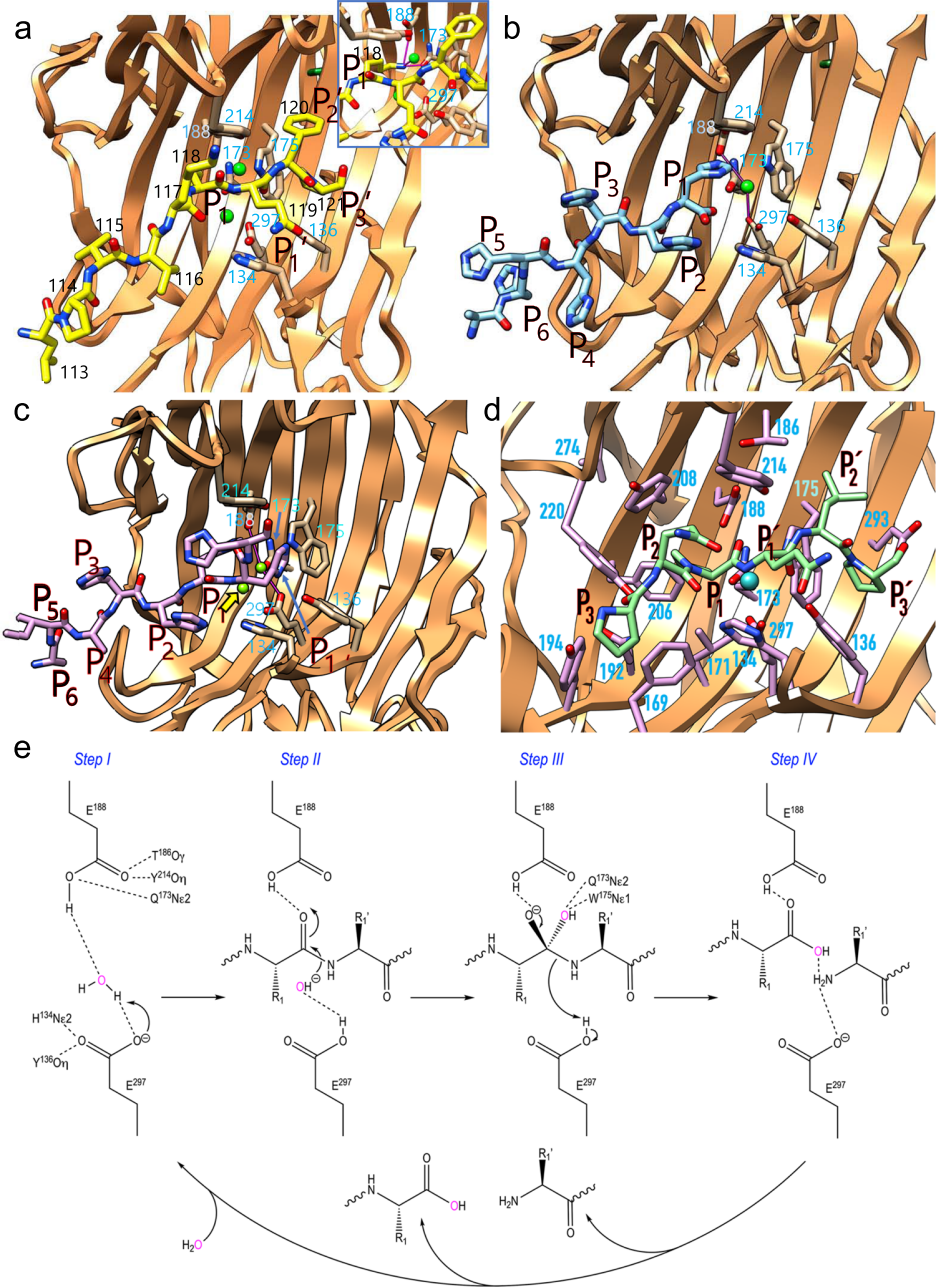
The active site and proposed mechanism. **a** Close-up of Fig. 5c depicting the final segment (L^113^–P^121^) of the PD defined in the final Fourier map as a stick model with yellow carbons and black residue numbers running across the active-site cleft. The likely **P**_**1**_ and **P**_**1**_**′**–**P**_**3**_**′** residues are labelled. In addition, selected residues of the active site are depicted for their side chains with carbons in tan and numbered in light blue. Two solvent residues potentially relevant for catalysis are shown as green spheres. The inset provides a slightly rotated close-up view to highlight the interaction (magenta lines) of K^118^ with E^188^, Q^173^ and a solvent molecule. **b** Same as (**a**) depicting the product complex of mature neprosin (crystal form I), with the C-terminal tail from a symmetry mate spanning A^403^ and the His_6_-tag residues (H^404^–H^409^) as a stick model with carbons in cyan featuring substrate subsites **P**_**6**_–**P**_**1**_. A solvent molecule potentially relevant for catalysis (green sphere) bridges E^297^ and E^188^ (magenta sticks). **c** Same as (**b**) for crystal form II. The C-terminal tail from a symmetry mate spanning A^401^ and part of the His_6_-tag (H^404^–H^408^) is shown as a stick model with carbons in plum, probably covering subsites **P**_**6**_–**P**_**1**_**′**. A solvent molecule potentially relevant for catalysis (green sphere) bridges E^297^ and E^188^ (magenta sticks). A second solvent molecule (yellow arrow) probably occupies the position of the scissile carbonyl oxygen in the Michaelis complex. The polypeptide chains of both crystal forms overlap for tag residues H^404^–H^409^ (crystal form I) and A^401^–H^406^ (crystal form II) upon superposition of the respective CDs. **d** Model of the likely Michaelis complex between a substrate spanning residues P–Q–P*Q–L–P (green carbons) at positions **P**_**3**_–**P**_**3**_**′** and the active site of neprosin. Selected residues are displayed for their side chain (plum carbons) and labelled. The catalytic solvent is depicted as a cyan sphere. **e** Proposed chemical mechanism of substrate cleavage by neprosin.

We hypothesized that the active-site cleft would be delineated by the PD linker (Fig. 6a) as found in other zymogens^42,44^. Moreover, in the structures of mature neprosin crystal forms I and II, the C-terminal segment, which spanned an alanine–isoleucine–alanine tripeptide followed by the His_6_-tag, ran along the surface of a symmetry mate, thus mimicking a product complex. Both crystal forms were monoclinic but with different cell constants (Supplementary Table 1), which resulted in variable crystal packing. Even so, the C-terminal tag penetrates the cleft in a similar manner in both crystallographic arrangements but is shifted by three positions, so that H^404^–H^409^ from crystal form I overlaps A^401^–H^406^ from crystal form II (Fig. 6b, c). Accordingly, neprosin would possess an extended active-site cleft traversing the concave face of the sheet, which is oblique to the direction of the front-sheet β-strands by ~55° (Fig. 6a–c).

On the search for possible catalytic residues, we were inspired by the functionally analogous pepsin-type aspartic peptidases, which despite their disparate architecture are likewise mainly β-proteins and operate at highly acidic pH^45^. Moreover, the only (weak) neprosin inhibitors we could find are also known to inhibit aspartate peptidases (see above). These enzymes use a pair of aspartic residues bridged by a solvent molecule for catalysis^46^. Indeed, we found a striking pair of glutamate residues (E^188^ and E^297^) bridged by a solvent molecule pinching the bound peptides in the product complexes (Fig. 6b, c). In crystal form II, a clearly resolved second solvent molecule would replace the scissile carbonyl oxygen of a substrate (Fig. 6c). The glutamate pair was similarly arranged in the zymogen structure, albeit slightly farther apart (Fig. 6a). We therefore produced E^188^Q, E^188^A, E^297^Q and E^297^A point mutants of His_6_-tagged pro-neprosin for testing (Fig. 1c). These variants did not autoactivate when incubated at acidic pH (Fig. 1f), so activation was triggered in trans using catalytic amounts of mature Strep-tagged wild-type neprosin. Finally, we obtained well-folded and intact mature variants of the E^188^Q and E^297^Q mutants, but not E^297^A or E^188^A (Fig. 1f), and these indeed were catalytically inactive (Fig. 2f). E^188^ and E^297^ may therefore act as a catalytic dyad, revealing that neprosin is a glutamate peptidase, a catalytic class that (in contrast to the aspartate peptidases) has been studied very poorly^47^. This is in agreement with very recent predictions based on bioinformatics studies but not validated experimentally^48^. Our results suggest that the mature neprosin structures mimic upstream product complexes collectively occupying subsites **S**_**6**_ to **S**_**1**_**′** (Fig. 6b, c), with the two catalytic glutamates plus the bridging solvent molecule poised for reaction. As the PD linker extends for further residues on the right side of the cleft in the zymogen (Fig. 6a), these would correspond to positions up to **P**_**3**_**′**. Thus, together with the extra space in the cleft beyond **S**_**3**_**′**, neprosin would feature an extended cleft, probably spanning up to 11 subsites (**S**_**6**_–**S**_**5**_**′**), which explains the need for extended peptides beyond the scissile bond (see above).

In the absence of a substrate complex, we constructed a model for the Michaelis complex of neprosin with the P–Q–P*Q–L–P peptide based on the zymogen and product–complex–mimicking structures (Fig. 6d). This model revealed further residues in the proximity of the catalytic glutamates with potential binding or catalytic functions. We therefore mutated residues H^134^, Y^136^, Q^173^, W^175^ and Y^214^ (Fig. 6a–d) by replacing them with alanine, and purified the corresponding proteins (Fig. 1c). All the mutants required activation in trans as discussed above (Fig. 1f). The activity of H^134^A, Y^136^A and Y^214^A was ~80% lower than the wild-type enzyme, whereas mutants Q^173^A and W^175^A were totally inactive (Fig. 2f). We conclude that H^134^, Y^136^ and Y^214^ are relevant but not critical for catalysis, possibly playing an ancillary role in the catalytic mechanism, whereas Q^173^ and W^175^ are essential (see below).

### Mechanisms of latency and activation

The PD attaches laterally to the left side of the mature enzyme so that its central β-sheet is rotated ~90° away from the plane of the front sheet. The inter-domain surface has a solvation-free energy gain upon interface formation (Δ^i^G) of –25.8 kcal/mol^49^, indicating a very strong interaction. Furthermore, the complex buries 2176 Å^2^, which exceeds the reported average value of 1910 Å^2^ for protein–protein complexes^50^. The PD (theoretical pI = 9.5; Fig. 5e, bottom) is crescent-shaped and snuggly embraces the CD (pI = 4.3; Fig. 5e, top) under electrostatic complementation, which contributes to activity repression and zymogen stability (pI = 5.9) at neutral or slightly acidic pH values. Moreover, the intimate zymogenic interaction further explains the remarkable stability of pro-neprosin in thermal shift assays (see above). Finally, the importance of the PD was further assessed by testing point mutant A^60^R, designed to destabilize the interface (Fig. 5c, left panel). This mutation prevented the isolation of a folded protein.

Once secreted to the acidic digestive fluid, the protonation of negatively charged residues leads to the repulsion of net positive charges so that the zymogen falls apart under liberation of the preformed mature moiety and the active-site cleft. The **S**_**1**_ position of the cleft is occupied by K^118^ from the PD linker in the zymogen structure, which was obtained at pH 7.5. This residue forms a strong salt bridge with catalytic E^188^ and a hydrogen bond with Q^173^Nε2, which is essential (see above and Fig. 6a, inset). We therefore produced and tested mutant K^118^A, which was efficiently overexpressed but underwent partial autolytic maturation in a neutral buffer, conditions under which the wild-type enzyme and other mutants remained intact (Fig. 1c). Subsequent incubation at pH 2.5 completed the activation process (Fig. 1f). As expected, the activity of the mature mutant was similar to that of the wild-type enzyme (Fig. 2f).

Based on the above, we propose that the K^118^–E^188^ pair features a latency plug that may be weakened once the zymogen reaches acidic environment by following a pH-switch mechanism, so the PD linker is pulled out for maturation cleavage. This is reminiscent of the digestive aspartate peptidases pepsin and gastricsin, which feature a lysine residue functionally equivalent to K^118^^43,51^, and of the lysosomal peptidase legumain^52^. The pH-switch mechanism, and the fact that the scissile **P**_**1**_**′**–**P**_**1**_ peptide bond is sandwiched by the Y^214^ side chain so that it is not accessible for cleavage (Fig. 6a), explains why the zymogen linker can bind in the direction of a substrate to the cleft at neutral pH without being cleaved. This contrasts with most zymogens, including digestive aspartate peptidases, in which pro-segments interact in a non-substrate-like manner with the mature enzyme residues as a mechanism to prevent untimely activation^42–44^. Finally, given that the scissile-bond position in the cleft is occupied by K^118^–Q^119^ but maturation occurs at P^128^–S^129^, activation probably occurs in trans by a second enzyme molecule once the PD linker is released from the cleft.

### Proposed catalytic mechanism

Based on the preceding results, the catalytic cleavage mechanism of neprosin would proceed as follows. The solvent bridging the E^188^ and E^297^ carboxylates in the product complexes would represent the catalytic water in the ground state (Fig. 6e, step I). The water is closer to E^297^, which suggests that E^188^ may be protonated, as reported for one of the two catalytic aspartates in pepsin-type acidic peptidases^46^. E^297^ is kept in place by hydrogen bonding with H^134^Nε2 and Y^136^Oη, whereas E^188^ is kept in place by hydrogen bonding with Y^214^Oη, T^186^Oγ and Q^173^Nε1. During the reaction, the substrate would bind to the active-site cleft in extended conformation (Fig. 6e, step II), with the **S**_**3**_, **S**_**1**_ and **S**_**3**_**′** subsites of the cleft being shaped by Y^194^, Q^192^ and F^169^; Y^208^, Y^206^, L^171^, E^188^ and Q^173^; and Y^136^, W^175^ and E^293^, respectively, which are ideal for the accommodation of prolines (Fig. 6d). The substrate main chain would be fixed by hydrogen bonds between its carbonyls and Y^220^Oη in **P**_**3**_, H^134^Nε2 in **P**_**2**_ (enabled by a 180º rotation around χ_2_ upon by substrate binding), and Y^136^Oη in **P**_**1**_**′** (Fig. 6d). Substrate insertion would shift the catalytic solvent further towards E^297^, which would act as a general base and abstract a proton from it to enhance its nucleophilicity. The protonated E^188^ carboxylate, in turn, would bind the scissile carbonyl oxygen (Fig. 6d, e). Thereafter, the polarized solvent would perform a nucleophilic attack on the si-face of the scissile carbonyl carbon, which would result in a tetrahedral gem-diolate reaction intermediate (Fig. 6e, step III). The latter would be stabilized by indispensable W^175^Nε1 and Q^173^Nε1, in the critical role of an oxyanion hole^53^. The intermediate would then resolve by breaking the scissile C–N bond. At this stage, E^297^ would act as a general acid and protonate the new α-amino nitrogen (Fig. 6e, step IV). Finally, the two cleavage products would leave the cleft and the enzyme would be poised for a new round of catalysis.

### Structural similarity with eqolysins

Peptidases were originally assigned to five mechanistic classes: the serine, cysteine, threonine, aspartate, and metal-dependent peptidases^54^. In 2004, the founding glutamate peptidase was structurally characterized, namely scytalidocarboxyl peptidase B (SCP-B) from the dematiaceous fungus *Scytalidium lignicolum*^55–57^. Since then, only the closely related aspergilloglutamic peptidase (~50% identical to SCP-B) has been structurally analysed^58,59^, and seven others have been functionally assessed, mostly from fungi^60–64^ but one from a bacterium^65^. They are assigned to family G1 in the MEROPS database and are informally known as the pepstatin-insensitive fungal carboxylpeptidase group^66^ or eqolysins^55^. They are thermophilic and pepstatin-insensitive enzymes that function under acidic conditions^65^ and feature a catalytic glutamate acting as a solvent-polarising general base, which is E_190_ in SCP-B (see UniProt ID P15369 for residue numbering in subscript according to the full-length protein, and subtract 54 for the commonly used mature enzyme numbering^55,56^). The glutamate is assisted by a glutamine (Q_107_ in SCP-B), hence the family name **eq**olysins^55^. These residues are invariant within the family and are flanked by very similar residues^66,67^.

**Fig. 7:**
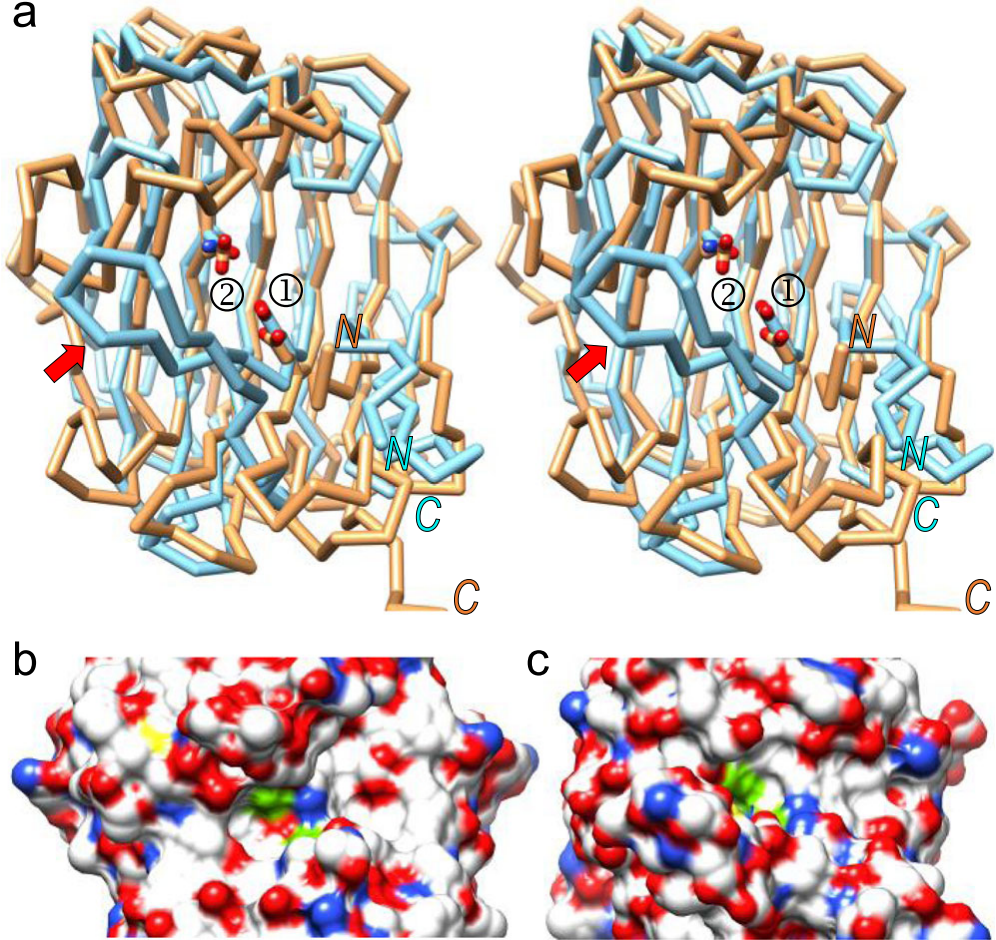
Structural similarity of neprosin and eqolysins. **a** Superposition of the Cα-traces of neprosin (salmon) and SCP-B (pale blue) in stereo, with the respective catalytic residues shown as sticks and labelled (➀, E^297^/E_190_ of neprosin/SCP-B; ➁, E^188^/Q_107_ of neprosin/SCP-B). Note the unique flap of SCP-B covering the active-site cleft (red arrow). The N-terminus and C-terminus are indicated. **b** Close-up of the active-site cleft of neprosin shown for its Connolly surface in the orientation of (**a**). The two catalytic residues are shown (green patches). **c** Same as (b) for SCP-B.

Archetypal SCP-B is a 7+7 antiparallel β-sandwich that shows overall similarity with the neprosin CD (Fig. 7a). Superposition of neprosin and the bound mature form of SCP-B (Protein Data Bank [PDB] ID 2IFR^56^), whose zymogenic structure is unknown, revealed 140 aligned residues with a rather large core RMSD of 3.0 Å and a sequence identity of only 11%. There are remarkable differences in the connecting loops and the active site, e.g. a large disulfide-linked protruding β-hairpin inserted in the fungal enzyme following the β-strand equivalent to β16 in neprosin (Fig. 7a). Within the active site, the only conserved residue is the catalytic glutamate (E^297^ in neprosin and E_190_ in SCP-B), as well as the position of the catalytic assistant (E^188^ in neprosin and Q_107_ in SCP-B), which lead to variable active-site clefts with disparate substrate trajectories and surface profiles (Fig. 7b, c). Moreover, cross-mutants Q_107_E of SCP-B and E^188^Q of neprosin, which mimic each other’s catalytic dyad, are completely inactive, as discussed above and reported in^68^. This explains the different substrate specificities, which in SCP-B leads to the cleavage of F–F, L–Y and F–Y bonds in insulin but not proline-flanking bonds^68^.

### Corollary

Current glutenases have limitations in meeting the stringent criteria for efficient oral enzyme therapy against CoD. Here, our in vitro and in vivo studies showed that recombinant neprosin is a robust pepsin-resistant enzyme that very efficiently degrades gliadin and its 33-mer under laboratory-simulated gastric conditions and in the mouse stomach. Low doses of the enzyme therefore complement gastric pepsin during digestion. Our results demonstrate that the Q–P*Q–L motif of the 33-mer is readily cleaved, which removes all six overlapping immunogenic epitopes by generating peptides too small to stimulate the division of gliadin-specific T cells^69^. The cleavage efficiency of neprosin in vitro under simulated gastric conditions is orders of magnitude higher than that of other glutenases^18,20,24,70–72^. The zymogen is produced at neutral pH, at which it remains stable and is lyophilizable for transport and storage. It only becomes activated after ingestion in the stomach and cleaves toxic components of gluten. Once the gastric bolus exits to the slightly acidic postprandial pH duodenum, it becomes inactive again. Neprosin is therefore a highly promising candidate for further therapeutic development against gluten-sensitive conditions.

Structural and functional studies backed by mutants and activity assays identified neprosin as a pepstatin-sensitive PEP and the only glutamate endopeptidase found in higher eukaryotes. It features a hitherto undescribed pair of catalytic glutamates that are analogous to the aspartates of the otherwise unrelated pepsin-type acidic endopeptidases. Neprosin is produced and secreted as a zymogen, which is activated only in its strongly acidic natural environment, the pitcher plant digestive fluid. Maturation follows a pH-switch mechanism that releases a lysine-mediated latency plug.

Finally, neprosin is pepstatin-sensitive but shares its overall fold with the pepstatin-resistant glutamate peptidases of the eqolysin family, which possess a glutamate–glutamine dyad and are represented by the archetype SCP-B. However, there are differences in the size of the PD and the CD, the active-site environment, the substrate-binding modus and specificity, as well as the chemical mechanism of catalysis. Furthermore, whereas eqolysins are restricted to fungi and bacteria^67,73^, potential neprosin orthologues with ~35–40% sequence identity are widely found in (and restricted to) plants, including gluten-containing crops. This suggests the neprosin family may have originated from a SCP-B ancestor by horizontal gene transfer from a bacterium or fungus to a plant, as previously described for other proteins^74^. Transfer would have been followed by divergent evolution within the plant kingdom to modify one of the catalytic residues and the loops decorating the central β-sandwich to adapt to new substrates. By analogy to the eqolysins, neprosin family members could be named eelysins.

## Methods

### Protein production and purification

A synthetic gene encoding wild-type neprosin from *Nepenthes × ventrata*, which is 91% identical to the orthologue from *Nepenthes alata* (UniProt ID A0A1L7NZU4), was inserted into vector pET-28a(+) by GenScript to produce vector pET-28a(+)-proNEP (for plasmids and primers, see Supplementary Table 2). The coding sequence was transferred to vector pCMV to produce vector pS6-proNEP. This conferred ampicillin resistance and added a C-terminal hexahistidine (His_6_) tag. The encoded protein is described herein as pro-neprosin. The same plasmid was modified by annealed oligonucleotide cloning to (a) replace the His_6_-tag with a twin Strep tag (pS6-proNEP-Strep) for the expression of pro-neprosin-strep, and (b) to remove the PD (pS6-NEP) for the expression of the neprosin CD (S^129^–Q^380^) plus the C-terminal His_6_-tag. The QuikChange Site-Directed Mutagenesis Kit (Stratagene) or inverse PCR-based site-directed mutagenesis were used to generate variants of pS6-proNEP with point mutations A^60^R, K^118^A, H^134^A, Y^136^A, Q^173^A, W^175^A, E^188^A, E^188^Q, Y^214^A, E^297^Q and E^297^A. Plasmids were purified with the GeneJET Plasmid MaxiPrep Kit (Thermo Fisher Scientific), and constructs were verified by DNA sequencing.

Proteins encoded by the pS6-proNEP, pS6-proNEP-Strep and pS6-NEP plasmids, as well as the 11 point mutants, were assessed for overexpression in human Expi293F cells (ThermoFisher Scientific) grown in a Multitron cell shaker incubator (Infors HT) at 37 °C. The cells were transfected with plasmid DNA and harvested after several days for protein purification. Cell-conditioned medium was cleared by centrifugation and supplemented with imidazole, incubated with nickel-nitrilotriacetic acid (Ni-NTA) resin (Invitrogen), subjected to batch affinity chromatography purification (AC), and washed extensively with buffer containing 20 mM imidazole. Proteins were eluted with the same buffer containing 300 mM imidazole. For pro-neprosin-strep, the Ni-NTA resin was replaced with Strep-Tactin XT Superflow suspension resin (IBA Life Sciences), and proteins were eluted in buffer containing 50 mM d-biotin (VWR Life Science). Fractions containing the protein were pooled and concentrated before size-exclusion chromatography (SEC) in a Superdex 75 10/300 GL column (GE Healthcare), which was attached to an ÄKTA Purifier liquid chromatography system (GE Healthcare).

Proteins were concentrated by ultracentrifugation in Vivaspin filter devices (Sartorius Stedim Biotech). Approximate protein concentrations were determined by measuring the absorbance at 280 nm (A_280_) using a BioDrop-DUO Micro Volume (Biochrom), and applying the appropriate theoretical extinction coefficients. Moreover, protein purity was assessed by sodium dodecylsulfate polyacrylamide gel electrophoresis (SDS-PAGE) followed by staining with Coomassie (Thermo Fisher Scientific). Protein identity was determined by peptide mass fingerprinting and N-terminal Edman sequencing at the Protein Chemistry Service and the Proteomics Facility of the Centro de Investigaciones Biológicas (Madrid, Spain), respectively. Finally, mature wild-type neprosin was lyophilized, stored at –20 °C, and reconstituted by dissolving in Milli-Q water.

For activity assays, the filtered conditioned medium of wild-type neprosin and the point mutants was supplemented with 3 mM reduced glutathione and 0.3 mM oxidized glutathione, the pH was adjusted with 20 mM Tris·HCl pH 8.0, and the mixture was incubated with cOmplete His-Tag Purification Resin (Roche). The resin was collected in an open column and the bound protein was washed with 10 mM Tris·HCl pH 7.0, 300 mM sodium chloride, and was then eluted with 100 mM glycine pH 2.5, 300 mM sodium chloride.

### Autolytic activation of pro-neprosin

Wild-type and mutant mature forms of neprosin or neprosin-strep were obtained by autolysis. Protein samples eluted from Ni-NTA or Strep-Tactin columns were dialysed against buffer, diluted twofold with 100 mM glycine pH 2.5, and incubated at 37 °C for up to 16 h. Reactions were stopped at specific time points (0 min, 10 min, 20 min, 30 min, 1 h, 2 h and overnight) by boiling aliquots in reducing/denaturing SDS sample buffer, followed by SDS-PAGE. Mature neprosin was buffer-exchanged to 20 mM Tris·HCl pH 7.5, 250 mM sodium chloride in a PD10 column followed by SEC in a Superdex 75 10/300 GL column with the same buffer. Protein purity and identity were assessed as stated above.

### Trans-activation of pro-neprosin mutants

To obtain mature neprosin point mutants from zymogens that do not autoactivate, the purified pro-proteins (H^134^A, Y^136^A, Q^173^A, W^175^A, E^188^A, E^188^Q, Y^214^A, E^297^Q and E^297^A) were incubated with activated neprosin-strep at a 20:1 weight ratio overnight at 37 °C. Pro-neprosin-strep was previously buffer-exchanged to 100 mM glycine pH 3.0, 150 mM sodium chloride for activation. Cleaved samples were buffer-exchanged and purified by reverse affinity chromatography, concentrated and purified by SEC.

### Protein stability assays

Pro-neprosin and mature neprosin were analysed by differential scanning fluorimetry using an iCycler iQ real-time PCR detection system (Bio-Rad). Samples were prepared at 0.5 mg/mL, in the presence or absence of 5 or 10 mM tris(2-carboxyethyl)phosphine (TCEP) as a reducing agent, and supplemented with 5× SYPRO Orange Protein Stain (Thermo Fisher Scientific). The temperature of midtransition (*T*_m_) was determined as the average of duplicate measurements of the midpoint value of the stability curve.

### Proteolytic activity and pH profile

We incubated 10 μM of the fluorescent protein substrate DQ Red BSA (Thermo Fisher Scientific) with 0.15 μM neprosin in 100 μL buffer at pH 2–8. Fluorescence was monitored using an Infinite M2000 microplate fluorimeter (Tecan) at 37 °C. We tested 0.5 μM bovine trypsin (Sigma-Aldrich) and porcine pepsin (Fluka) for comparison. Each assay was carried out in triplicate.

### Cleavage studies with fluorogenic peptides and determination of kinetic parameters

The kinetic parameters of FS6-QPQL peptide (Mca–Q–P–Q–L–Dpa–A–R–NH_2_; GenScript) cleavage by wild-type neprosin (25 nM final enzyme concentration), as well as those of the FS6 peptide (Mca–K–P–L–G–L–Dpa–A–R–NH_2_; Sigma-Aldrich) by neprosin at 100 nM final enzyme concentration, were determined in reactions containing 100 mM glycine pH 3.0 and substrate concentrations of 1–75 μM (FS6-QPQL) or 2.5–75 μM (FS6) at 37 °C. The fluorescence signal, representing cleavage product formation, was recorded over time for each substrate concentration and the initial rate (*v*_0_) was derived from the slope of the linear part of the curve. Using a range of substrate concentrations and a surplus of peptidase, we measured the fluorescence signal generated after full substrate turnover and calculated the corresponding fluorescence units per picomole of cleaved substrate. These values were plotted against substrate concentration and fitted to the hyperbolic Michaelis-Menten equation (*v* = *V*_max_·[S]/{*K*_M_+[S]}) by nonlinear regression using *GraphPad*^75^ and *SigmaPlot*^76^ to determine the maximum velocity (*V*_max_), the Michaelis substrate affinity constant (*K*_M_), the turnover rate (*k*_cat_ = *V*_max_/[E_total_]), and the catalytic efficiency (*k*_cat_/*K*_M_) of the cleavage reaction. All experiments were carried out in triplicate.

The peptidolytic activity of wild-type neprosin was compared to that of the mutants K^118^A, H^134^A, Y^136^A, Q^173^A, W^175^A, E^188^Q, Y^214^A and E^297^Q (140 ng) using 10 μM of the fluorogenic FS6-QPQL peptide in 100 mM glycine pH 3.0, 150 mM sodium chloride at 37 °C, shaking in a Synergy H1 microplate reader (BioTek). To ensure identical sample treatment, all protein variants were activated with neprosin-strep, which was then removed by reverse affinity chromatography as stated above. The protein concentration was estimated from the surface of the A_280_ SEC curves and corrected based on the ε_280_ values. Fluorescence values after 30 min were used as activity endpoints. Experiments were carried out in triplicate and differences were analysed for statistical significance using *GraphPad*.

### Cleavage of gliadin in vitro

Wheat gliadin (Sigma-Aldrich) was prepared in 100 mM glycine pH 2.5 and variable concentrations of pepsin (0.05–10 μM) from porcine gastric mucosa (Fluka), neprosin (0.05–2 μM), or mixtures of 0.5 μM pepsin and 0.05–2 μM neprosin were used to digest 10 mg/mL gliadin slurries. Reactions were monitored by turbidimetry in 96-well plates (Corning) at 37 °C in a microplate spectrophotometer (BioTek). Reactions were quenched by boiling in SDS sample buffer before analysis by SDS-PAGE. Gliadin degradation by neprosin was also analysed by zymography using SDS-PAGE gels containing either wheat gliadin or teleostean gelatin (Sigma-Aldrich), which was used as a control, at 0.1 mg/mL. Pro-neprosin was also tested, which became activated to the mature form during the assay. Proteins were renatured by washing the zymograms with 2.5% Triton X-100 in 100 mM glycine pH 2.5, 200 mM sodium chloride. After further washes with the same buffer plus 0.02% Brij-35, the zymograms were incubated overnight in the same buffer, rinsed briefly with water, and stained with Coomassie.

### Cleavage of the 33-mer peptide in vitro

Cleavage of the 33-mer peptide of wheat α-gliadin (LQLQPFPQPQLPYPQPQLPYPQPQLPYPQPQPF, 3911 Da) was monitored using an AutoFLEX III MALDI-TOF mass spectrometer. The peptide (from GenScript) was dissolved in water to a concentration of ~20 mg/mL and stored at –20 °C. The cleavage reaction was carried out with ~1 mg/mL (~250 μM) substrate in 100 mM glycine pH 3.0 at 37 °C by adding 0.5 μM neprosin or 10 μM pepsin. Reactions were stopped at different time points (0 min, 10 min, 20 min, 45 min, 1 h and overnight) and samples were then diluted 1:10 with water, mixed with an equal volume of the 2,5-dihydroxybenzoic acid matrix at 10 mg/mL in a solution containing 30% acetonitrile and 70% 0.1% trifluoroacetic acid, and spotted on a ground steel plate (Bruker). Mass spectra were acquired in positive reflectron mode at 21 kV total acceleration voltage.

### Liquid chromatography-mass spectrometry (LC-MS/MS) data analysis

We reanalysed the cleavage specificity data of endogenous neprosin or recombinant material obtained from *Escherichia coli* deposited at Chorus (Project ID 1262^25^). LC-MS/MS raw files were converted to MGF format, and data were processed using *TANDEM*, *Comet* and *MS-GF+*, as implemented in *SearchGUI*^77^. Results were evaluated using *PeptideShaker*^78^ with a false discovery rate of 1%. Data were non-specifically searched for hits against the human proteome in UniProt (March 2020) using a mass tolerance of 20 ppm for both MS1 and MS2, fixed cysteine carbamidomethylation, and variable methionine oxidation. Up to 50 missed cleavages or a maximum of 5500 Da were tolerated for the parental peptide mass.

### Inhibition assays

On the search for neprosin inhibitors, we assayed the broad-spectrum cOmplete Inhibitor Cocktail (Roche); the metallopeptidase inhibitors 1,10-phenathroline, phosphoramidon, marimastat, and captopril (all from Sigma-Aldrich); the serine peptidase inhibitor 4-(2-aminoethyl)-benzenesulfonyl fluoride (AEBSF; Sigma-Aldrich); the aspartate peptidase inhibitors pepstatin A (Sigma-Aldrich), methyl-2-[(2-diazoacetyl)amino]hexanoate (DAN; Chemical Abstracts Service (CAS) 7013-09-4; Bachem 4010441), and ENPN (CAS 5255-75-4; Apollo Scientific OR26560); as well as the proline-containing/mimicking compounds 2-acetyl-1-methylpirrole (AMP; CAS 932-16-1; Sigma-Aldrich 160865); (*S*)-*tert*-butyl-2-(3-ethoxy-3-oxopropanoyl)pyrrolidine-1-carboxylate (BEOPC; CAS 109180-95-2; Fluorochem 387901); and *N*-boc-glycylproline (BGP; CAS 14296-92-5; Bachem 4003703). Inhibition of the cleavage of the FS6-QPQL peptide was investigated by pre-incubating 100 nM neprosin in 100 mM glycine pH 3.0 with 100 μM of each tester compound for >1 h at 37 °C. We then added 10 μM of the substrate and the residual activity was monitored for 4 h as an increase in fluorescence. Differences were analysed for statistical significance using *GraphPad*. The positive control in the absence of inhibitors (100% activity) contained the same final concentration of dimethyl sulfoxide that was used to solubilize the inhibitors. In addition, half-maximal inhibitory concentration (IC_50_) values were determined for pepstatin A and ENPN by measuring the activity of 50 nM neprosin in the presence of 10 μM of substrate and inhibitor concentrations of 5–500 μM and 5–5000 μM, respectively, to obtain the inhibition curves. These curves were analysed by nonlinear regression using *GraphPad*.

### Evaluation of gliadin processing by neprosin in vivo

Experimental procedures involving mice followed the institutional guidelines for the care and use of laboratory animals and the ARRIVE guidelines. Protocols were approved by the Ethical Committee for Animal Experimentation of the University of Barcelona (CEEA-UB/Ref. 186/20-P2) and the Government of Catalonia (PAMN/Ref. 11485), which followed Directive 2010/63/EU for the protection of animals used for scientific purposes. The sample size was estimated by the Appraising Project Office’s program from the Universitat Miguel Hernández of Elx (Alacant, Spain). We used 5-week-old male and female C57BL/6 mice (n = 16) purchased from Janvier and housed at the animal facility of the Faculty of Pharmacy and Food Science of the University of Barcelona in a controlled environment (20–24 °C, 40–60% relative humidity) and a 12-h photoperiod, with lights on at 8 a.m. and lights off at 8 p.m. Animals were housed in cages with large Souralit 1035 fibrous particles as bedding (Bobadeb), and tissue paper (Gomà-Camps) and cardboard climbing structures for cage enrichment. Animals had free access to water and RM3 (P) SQC diet (Special Diet Services).

After 1 week for acclimation, two groups of mice were randomly selected, each comprising four males and four females (*n* = 8 per group) and were marked neprosin (N) or vehicle (V). Animals were not fasted to account for the physiological transit time, and food and water were removed only 1 h before oral gavage. Group N mice were fed 50 μL pro-neprosin in vehicle (0.2 mg/mL in 20 μM Tris-buffered saline pH 7.5, 150 μM sodium chloride), whereas group V mice were fed 50 μL vehicle alone. After 5 min, all mice were fed 50 μL gliadin slurry containing 5 mg wheat gliadin (Sigma-Aldrich) at 100 mg/mL in 10% ethanol solution using small-volume Hamilton syringes and adapted oral probes. The enzyme:gliadin ratio (1:500) was calculated based on our in vitro results, which had shown that neprosin digests gliadin at a 1:500–1000 ratio at 37 °C over a period of 90 min. Given that gastrointestinal transit in mice causes a bolus to reach the small intestine after 1–3 h, with some content already entering the large intestine^79^, we selected 2.5 h as the optimal endpoint to assess the degradation of gliadin in the upper gastrointestinal tract. Animals were then euthanized by cervical dislocation and the contents of the stomach, proximal small intestine and distal small intestine were removed, weighed, and frozen at –20 °C.

Samples were suspended in phosphate-buffered saline (pH 7.2) at a concentration of 200 mg/mL, homogenized with a Kimble Pellet Pester Cordless Motor (DWK Life Sciences), and extracted first with buffer at 50 °C for 40 min and then with 80% ethanol at 20–25 °C for 1 h. The mixtures were centrifuged (2000 × *g*, 10 min, 4 °C), and the aqueous layer between the particulate and fat layers was removed. The 33-mer content in each diluted extract was analysed using the AgraQuant Gluten G12 ELISA test kit (Romer Labs), which has a detection limit of 2 ppm, according to the manufacturer’s instructions. The G12 antibody detects the 33-mer but no other gliadin degradation fragments^80^. Final amounts were normalized taking into account the sample weight and results were expressed as mean ± SEM. The *Statistical Package for Social Sciences* (SPSS v22.0; IBM) was used for statistical analysis. The data showed homogeneity of variance (Levene’s test) and followed a normal distribution (Shapiro-Wilk test), so we applied conventional one-way analysis of variance (ANOVA).

### Crystallization and diffraction data collection

We screened for crystallization conditions at the joint IBMB/IRB Automated Crystallography Platform using the sitting-drop vapour diffusion method. Optimal pro-neprosin crystals (~20 mg/mL in 20 mM Tris·HCl pH 7.5, 150 mM sodium chloride) were obtained at 20 °C with 0.1 M sodium acetate pH 4.0, 22% polyethylene glycol (PEG) 6000, 10% isopropanol as the reservoir solution. Crystals were harvested using cryo-loops (Molecular Dimensions), rapidly passed through a cryo-buffer consisting of reservoir solution plus 15% (v/v) glycerol, and flash-vitrified in liquid nitrogen for data collection. A lutetium derivative of pro-neprosin was obtained by soaking native crystals for 5 min in cryo-buffer supplemented with 100 mM of the Lu-Xo4 crystallophore (Polyvalan)^40^ and flash-vitrifying them without back soaking. X-ray diffraction data were collected from native crystals at 100 K on a Pilatus 6M-F pixel detector at beamline I04-1 of the Diamond Light Source (Harwell, UK). Lutetium derivative data were recorded on a Pilatus 6M detector at beamline XALOC of the ALBA synchrotron (Cerdanyola, Catalonia, Spain) operated with the *Generic Data Acquisition* (GDA) software.

The mature neprosin–product complex (crystal form I) was obtained at a protein concentration of ~16 mg/mL in 20 mM Tris·HCl pH 7.5, 250 mM sodium chloride at 4 °C using 10% PEG 1000, 10% PEG 8000 as the reservoir solution. Crystals were cryo-protected with the same reservoir solution plus 15% (v/v) glycerol prior to flash-vitrification in liquid nitrogen. X-ray diffraction data at 100 K were collected at beamline ID30B of the ESRF synchrotron (Grenoble, France) using a Pilatus 6M detector. The mature neprosin–product complex in crystal form II was obtained at the same protein concentration but in 0.1 M glycine pH 3.0, 150 mM sodium chloride at 20 °C using 0.1 M sodium citrate tribasic pH 5.6, 0.5 M ammonium sulfate, 1 M lithium sulfate as the reservoir solution. Crystals were cryo-protected with a solution containing 20% (v/v) glycerol. Diffraction data were collected at beamline XALOC on a Pilatus 6M detector.

Diffraction data were processed using *Xds*^81^ and *Xscale*, and were transformed to MTZ format using *Xdsconv* for the *Phenix*^82^ and *Ccp4*^83^ program suites. All crystals contained a monomer in the crystal asymmetric unit and Supplementary Table 1 provides essential statistics on data collection and processing.

### Structural solution and refinement

The structure of pro-neprosin was solved by single-wavelength anomalous diffraction using data collected from a lutetium derivative crystal at the L_III_-absorption peak wavelength (1.34 Å) by applying the *Autosol* protocol of the *Phenix* package. The resulting Fourier map was then subjected to further density modification with *wARP/ARP*^84^. A starting model for Lu-Xo4 was obtained by energy minimization applied to the coordinates of the metal-chelating moiety of the compound as found in its complex with Tb^3+^ (Protein Data Bank [PDB] ID 6FRO, residue name 7MT) using *Chimera*^85^. The resulting coordinates in PDB format were combined with a Lu^3+^ ion for model building. Thereafter, several rounds of manual model building in *Coot*^86^ alternated with crystallographic refinement using the *Refine* protocol of *Phenix* and the *BUSTER*^87^ program. The final model comprised pro-neprosin residues A^29^–Q^380^ except S^76^–Y^85^ and N^122^–N^131^ plus three extra C-terminal residues from the purification tag (A^401^–I^402^–A^403^); two Lu-Xo4 moieties at roughly half occupancy; two *N*-linked glycan chains totalling five sugar residues attached to N^145^ and N^152^, respectively; two acetate molecules; and 180 solvent molecules.

The structure of native pro-neprosin was solved by molecular replacement using the *Phaser* crystallographic software within *Ccp4* and the protein coordinates of the lutetium derivative crystal structure. Subsequent model building and refinement proceeded as described above. The final model included residues A^29^–Q^380^ except Y^77^–Y^85^ and N^122^–N^131^ plus two extra C-terminal residues from the purification tag (A^401^–I^402^), two *N*-linked glycan chains totalling four sugar residues, as well as eight acetate, one isopropanol, four glycerol and 257 solvent molecules.

The structure of a product complex of native mature neprosin in crystal form I was also solved by molecular replacement, using the coordinates of fragment T^132^–I^402^ from native pro-neprosin. Subsequent model building and refinement proceeded as described above. The final model spanned residues T^132^–Q^380^ plus the entire C-terminal tag (A^401^–I^402^–A^403^+H^404^–H^409^), two *N*-linked glycan chains totalling seven sugar residues, plus one triethylene glycol and 171 solvent molecules. The structure of a product complex of native mature neprosin in crystal form II was also solved by molecular replacement, using fragment T^132^–Q^380^ of the crystal form I complex. The final model included residues T^132^–Q^380^ plus the C-terminal tag except H^409^ (A^401^–I^402^–A^403^+H^404^–H^408^), as well as two *N*-linked glycan chains totalling four sugar residues plus one nickel cation, three sulfate anions, one tetraglycine and one glycine, as well as 250 solvent molecules. The nickel ion, presumably from the Ni-NTA resin used for purification, was tentatively assigned based on short liganding distances to two histidine residues (~1.8 Å), which were closer to those reported for tetrahedrally-coordinated nickel ions (1.88 Å on average) than to those of the more abundant lithium (2.03 Å) from the reservoir solution^88^. A tetraglycine was tentatively placed in an adequate density region based on the capacity of this amino acid to oligomerize under certain conditions^89^. Supplementary Table 1 provides essential statistics on the final refined models, which were validated using the *wwPDB Validation Service* at https://validate-rcsb-1.wwpdb.org/validservice and deposited with the PDB at www.pdb.org (access codes 7ZU8, 7ZVA, 7ZVB and 7ZVC).

### Miscellaneous

Structural superpositions and structure-based sequence alignments were calculated using the *SSM* program within *Coot*. Figures were prepared using *Chimera*. Structure-based similarity searches were performed with *Dali*^90^. Protein interfaces were calculated with *PDBePISA* at www.ebi.ac.uk/pdbe/pisa. The interacting surface of a complex was defined as half the sum of the buried surface areas of either molecule.

### Reporting summary

Further information on research design is available in the Nature Research Reporting Summary linked to this article.

## Supplementary information

Supplementary Information

Reporting Summary

## Data Availability

All data and reagents are freely available from the authors upon reasonable request and signature of non-disclosure and material transfer agreements for non-profit usage by academic groups. Source data are provided with this paper. Atomic coordinates are available from the Protein Data Bank under codes 7ZU8, 7ZVA, 7ZVB and 7ZVC. Source data are provided with this paper.

## Source data

Source Data
