# Supplementary material for: Molecular and in vivo studies of a glutamate-class prolyl-endopeptidase for coeliac disease therapy
Source: Nat Commun. 2022 Aug 1;13:4446. doi: 10.1038/s41467-022-32215-1 (PMC9343461; doi:10.1038/s41467-022-32215-1)
Supplement: Supplementary file 1 — Supplementary Information [file 41467_2022_32215_MOESM1_ESM.pdf]

## SUPPLEMENTARY INFORMATION

# Molecular and *in vivo* studies of a glutamate-class prolyl-endopeptidase for coeliac disease therapy

Laura del Amo-Maestro, Soraia R. Mendes, Arturo Rodríguez-Banqueri, Laura Garzon-Flores, Marina Girbal, María José Rodríguez-Lagunas, Tibisay Guevara, Àngels Franch, Francisco J. Pérez-Cano, Ulrich Eckhard and F. Xavier Gomis-Rüth

**Supplementary Table 1. Crystallographic data.**

| ***Dataset*** | **Pro-neprosin** ***Lu-Xo4 complex*** | **Pro-neprosin** ***Native*** | **Neprosin** ***Product complex I*** | **Neprosin** ***Product complex II*** |
|---|---|---|---|---|
| Beam line (synchrotron) | XALOC (ALBA) | I04-1 (DIAMOND) | ID30B (ESRF) | XALOC (ALBA) |
| Space group / protomers per a.u. [a] | $P2_12_12$ / 1 | $P2_12_12$ / 1 | $P2_1$ / 1 | $P2_1$ / 1 |
| Cell constants (a, b, c, in Å; β in º) | 86.65, 93.35, 48.70, 90.0 | 86.37, 92.62, 42.69, 90.0 | 54.76, 49.95, 59.86, 106.57 | 58.86, 40.63, 64.63, 107.98 |
| Wavelength (Å) | 1.3400 | 1.0050 | 1.0050 | 0.97926 |
| Measurements / unique reflections | 316,335 / 25,470 [f] | 407,612 / 32,321 | 84,480 / 13,077 | 160,050 / 24,437 |
| Resolution range (Å) (outermost shell) [b] | 63.5 – 2.05 (2.17 – 2.05) | 63.2 – 1.80 (1.91 – 1.80) | 52.5 – 2.35 (2.49 – 2.35) | 61.5 – 1.85 (1.96 – 1.85) |
| Completeness (%) / $R_{merge}$ [c] | 99.9 (99.4) / 0.182 (1.493) | 99.3 (96.0) / 0.110 (1.552) | 99.6 (99.2) / 0.235 (1.167) [g] | 97.1 (86.8) / 0.127 (0.941) |
| $R_{meas}$ [c] / CC(1/2) [c] | 0.190 (1.564) / 0.997 (0.918) | 0.115 (1.634) / 0.999 (0.710) | 0.256 (1.270) [g] / 0.990 (0.653) | 0.138 (1.040) / 0.998 (0.729) |
| Average intensity [d] | 9.2 (2.4) | 15.3 (1.5) | 5.6 (1.3) | 12.4 (2.0) |
| B-Factor (Wilson) ($Å^2$) / Aver. multiplicity | 39.8 / 12.5 (11.4) | 34.4 / 12.6 (10.3) | 40.3 / 6.5 (6.4) | 26.0 / 6.5 (5.6) |
| Heavy-ion sites for phasing | 2 | – | – | – |
| | | | | |
| Resolution range used for refinement (Å) | 63.5 – 2.05 | 23.2 – 1.80 | 52.5 – 2.35 | 20.7 – 1.85 |
| Reflections used (test set) | 24,846 (621) | 31,666 (635) | 12,598 (479) | 23,726 (690) |
| Crystallographic $R_{factor}$ (free $R_{factor}$) [c] | 0.209 (0.250) | 0.182 (0.218) | 0.195 (0.246) | 0.173 (0.203) |
| Non-H protein atoms / ionic ligands / | 2613 / 2 ACT, 2 Lu-Xo4 | 2608 / 8 ACT | 2011 / – / | 1999 / 1 $Ni^{2+}$, 3 $SO_4^{2-}$/ |
| waters / non-ionic ligands per a.u. | 179/1 FUC,4 NAG | 256/1 FUC, 5 GOL,3 NAG,1 IPA | 171/1 BMA,3 MAN,3 NAG, 2 PGE | 250/1 BMA,3 NAG,1 $GLY_4$,1 GLY |
| *Rmsd* from target values | | | | |
| bonds (Å) / angles (°) | 0.007 / 0.96 | 0.008 / 0.99 | 0.008 / 1.02 | 0.011 / 1.07 |
| Average B-factor ($Å^2$) | 48.5 | 35.8 | 39.6 | 27.9 |
| Analysis of protein contacts and geometry [e] | | | | |
| Ramachandran favoured / outliers / all analyzed | 317 (95.8%) / 1 (0.3%) / 331 | 321 (95.8%) / 1 (0.3%) / 335 | 246 (96.0%) / 1 (0.4%) / 256 | 250 (96.5%) / 1 (0.4%) / 259 |
| Bond-length/ bond-angle / chirality / plan. outliers | 0 / 0 / 0 / 1 | 0 / 0 / 0 / 1 | 0 / 0 / 0 / 0 | 0 / 0 / 0 / 1 |
| Side-chain outliers | 10 (3.6%) | 7 (2.5%) | 7 (3.4%) | 1 (0.5%) |
| All-atom clashes / clashscore [e] | 13 / 2.4 | 11 / 2.0 | 9 / 2.1 | 4 / 1.0 |
| RSRZ outliers [e] / $F_o$:$F_c$ correlation | 23 (6.8%) / 0.91 (0.89) | 17 (5.1%) / 0.95 (0.93) | 1 (0.4%) / 0.93 (0.90) | 3 (1.1%) / 0.95 (0.94) |
| PDB access code | 7ZU8 | 7ZVA | 7ZVB | 7ZVC |

[a] Abbreviations: ACT, acetate; a.u., crystallographic asymmetric unit; BMA, β-d-mannose; IPA, isopropanol; GLY, glycine; $GLY_4$, tetraglycine; FUC, α-l-fucose; GOL, glycerol; MAN, α-d-mannose; NAG, β-*N*-acetyl-d-glucosamine; PGE, triethylene glycol; RSRZ, real-space R-value Z-score. [b] Values in parentheses refer to the outermost resolution shell. [c] For definitions, see [1]. [d] Average intensity is $< I/\sigma(I) >$ of unique reflections after merging according to *Xscale* [2]. [e] According to the wwPDB Validation Service (https://wwpdb-validation.wwpdb.org/validservice). [f] Friedel mates were kept separate for structural solution but merged for refinement. [g] The overall high $R_{meas}$ and $R_{merge}$ values of the only crystal obtained of this crystal form resulted from insufficient exposure times, which led to weak measurements.

**Supplementary Table 2. Constructs and primers for neprosin expression in mammalian cells.**

| Construct name | Parental plasmid | Forward primer | Reverse primer | Protein sequence |
|---|---|---|---|---|
| pS6-proNEP-Strep | pS6-proNEP | CGCTGGAGGTTGGAGCCATCCACAATTCGAAAAGGGTGGAGGTTCTGGAGGTGGAAGTGGAGGTTGGAGCCATCCACAATTCGAAAAGTAGTAGC | TCGAGCTACTACTTTTCGAATTGTGGATGGCTCCAACCTCCACTTCCACCTCCAGAACCTCCACCCTTTTCGAATTGTGGATGGCTCCAACCTCCAGCGAT | DLMV+R$^{25}$–Q$^{380}$+AIA–GGWSHPQFEKGGGSGGGSGGWSHPQFEK |
| pS6-proNEP | pET-28a(+)–proNEP * | ATGCGGTGACCTAATGGTACGTAGCATTCA | GCATGCGATCGCTTGGCAACCCGGACCA | DLMV+R$^{25}$–Q$^{380}$+AIA–H$_6$ |
| pS6-NEP | pS6-proNEP | ATGCGGTGACCTAAGCGCGAACACCAACCA | GCATGCGATCGCTTGGCAACCCGGACCAC | DLMV+S$^{129}$–Q$^{380}$+AIAH$_6$ |
| **Point mutants** | | | | |
| pS6-proNEP-A$^{60}$R | pS6-proNEP | TCTACAAGCAGCCGAGGTTCGATCACCC | GGGTGATCGAACCTCGGCTGCTTGTAGA | DLMV+R$^{25}$–A$^{60}$R–Q$^{380}$+AIA–H$_6$ |
| pS6-proNEP-K$^{118}$A | pS6-proNEP | CGGTGGTTAAGGCACAATTCCCGAACCT | AGGTTCGGGAATTGTGCCTTAACCACCG | DLMV+R$^{25}$–K$^{118}$R–Q$^{380}$+AIA–H$_6$ |
| pS6-proNEP-Y$^{136}$A | pS6-proNEP | ACCAACCACCAGGCTGCGGTTATTGCGT | ACGCAATAACCGCAGCCTGGTGGTTGGT | DLMV+R$^{25}$–Y$^{136}$R–Q$^{380}$+AIA–H$_6$ |
| pS6-proNEP-E$^{188}$A | pS6-proNEP | CCTGAACACCATTGCAGCGGGTTGGCAG | CTGCCAACCCGCTGCAATGGTGTTCAGG | DLMV+R$^{25}$–E$^{188}$A–Q$^{380}$+AIA–H$_6$ |
| pS6-proNEP-E$^{188}$Q | pS6-proNEP | CCTGAACACCATTCAAGCGGGTTGGCAG | CTGCCAACCCGCTTGAATGGTGTTCAGG | DLMV+R$^{25}$–E$^{188}$Q–Q$^{380}$+AIA–H$_6$ |
| pS6-proNEP-Y$^{214}$A | pS6-proNEP | GACCGCGGACGGTGCTACCAGCACCG | CGGTGCTGGTAGCACCGTCCGCGGTC | DLMV+R$^{25}$–Y$^{214}$A–Q$^{380}$+AIA–H$_6$ |
| pS6-proNEP-E$^{297}$A | pS6-proNEP | GAGTGGGGTGGCGCAATCTACGATAGCA | TGCTATCGTAGATTGCGCCACCCCACTC | DLMV+R$^{25}$–E$^{297}$A–Q$^{380}$+AIA–H$_6$ |
| pS6-proNEP-E$^{297}$Q | pS6-proNEP | GAGTGGGGTGGCCAAATCTACGATAGCA | TGCTATCGTAGATTTGGCCACCCCACTC | DLMV+R$^{25}$–E$^{297}$Q–Q$^{380}$+AIA–H$_6$ |

* A cDNA encoding pro-neprosin inserted into *Escherichia coli* plasmid pET-28a(+) was purchased from GeneScript. Restriction-site sequences are underlined, amino acid point mutations are in magenta, additional residues derived from the cloning strategy are in red, C-terminal tags are in blue.

## SUPPLEMENTARY REFERENCES


1 Einspahr, H. M. & Weiss, M. S. in *International Tables for Crystallography. Volume F: Crystallography of biological macromolecules.* (eds E. Arnold, D.M. Himmel, & M.G. Rossmann) 64-74 (John Wiley & Sons, Inc., 2012).

2 Kabsch, W. XDS. *Acta Crystallogr. sect. D* **66**, 125-132, doi:10.1107/S0907444909047337 (2010).
