# Supplementary material for: Molecular and in vivo studies of a glutamate-class prolyl-endopeptidase for coeliac disease therapy
Source: Nat Commun. 2022 Aug 1;13:4446. doi: 10.1038/s41467-022-32215-1 (PMC9343461; doi:10.1038/s41467-022-32215-1)
Supplement: Supplementary file 2 — Reporting Summary [file 41467_2022_32215_MOESM2_ESM.pdf]

nature portfolio

Corresponding author(s): F. Xavier Gomis-Rüth

Last updated by author(s): Jul 8, 2022

Reporting Summary

Nature Portfolio wishes to improve the reproducibility of the work that we publish. This form provides structure for consistency and transparency in reporting. For further information on Nature Portfolio policies, see our Editorial Policies and the Editorial Policy Checklist.

Statistics

For all statistical analyses, confirm that the following items are present in the figure legend, table legend, main text, or Methods section.

n/a	Confirmed	
☐	☒	The exact sample size (*n*) for each experimental group/condition, given as a discrete number and unit of measurement
☐	☒	A statement on whether measurements were taken from distinct samples or whether the same sample was measured repeatedly
☐	☒	The statistical test(s) used AND whether they are one- or two-sided<br>*Only common tests should be described solely by name; describe more complex techniques in the Methods section.*
☒	☐	A description of all covariates tested
☒	☐	A description of any assumptions or corrections, such as tests of normality and adjustment for multiple comparisons
☐	☒	A full description of the statistical parameters including central tendency (e.g. means) or other basic estimates (e.g. regression coefficient) AND variation (e.g. standard deviation) or associated estimates of uncertainty (e.g. confidence intervals)
☐	☒	For null hypothesis testing, the test statistic (e.g. *F*, *t*, *r*) with confidence intervals, effect sizes, degrees of freedom and *P* value noted<br>*Give P values as exact values whenever suitable.*
☒	☐	For Bayesian analysis, information on the choice of priors and Markov chain Monte Carlo settings
☒	☐	For hierarchical and complex designs, identification of the appropriate level for tests and full reporting of outcomes
☒	☐	Estimates of effect sizes (e.g. Cohen's *d*, Pearson's *r*), indicating how they were calculated

Our web collection on statistics for biologists contains articles on many of the points above.

Software and code

Policy information about availability of computer code

Data collection	MxCuBE, Beamline Expert System (BES), ExiMX, Generic Data Acquisition (GDA)
Data analysis	XDS, XSCALE, XDSCONV, FAST_DP, XIA2, PHENIX

For manuscripts utilizing custom algorithms or software that are central to the research but not yet described in published literature, software must be made available to editors and reviewers. We strongly encourage code deposition in a community repository (e.g. GitHub). See the Nature Portfolio guidelines for submitting code & software for further information.

Data

Policy information about availability of data

All manuscripts must include a data availability statement. This statement should provide the following information, where applicable:

- Accession codes, unique identifiers, or web links for publicly available datasets
- A description of any restrictions on data availability
- For clinical datasets or third party data, please ensure that the statement adheres to our policy

All data and reagents are freely available from the authors upon reasonable request and signature of non-disclosure and material transfer agreements for non-profit usage by academic groups. The PDB coordinates of the structures solved in this work have been deposited with the Protein Data Bank. Source data are provided with this paper. Atomic coordinates are available from the Protein Data Bank under codes 7ZU8, 7ZVA, 7ZVB and 7ZVC.

Human research participants

Policy information about studies involving human research participants and Sex and Gender in Research.

Reporting on sex and gender	*Use the terms sex (biological attribute) and gender (shaped by social and cultural circumstances) carefully in order to avoid confusing both terms. Indicate if findings apply to only one sex or gender; describe whether sex and gender were considered in study design whether sex and/or gender was determined based on self-reporting or assigned and methods used. Provide in the source data disaggregated sex and gender data where this information has been collected, and consent has been obtained for sharing of individual-level data; provide overall numbers in this Reporting Summary. Please state if this information has not been collected. Report sex- and gender-based analyses where performed, justify reasons for lack of sex- and gender-based analysis.*
Population characteristics	*Describe the covariate-relevant population characteristics of the human research participants (e.g. age, genotypic information, past and current diagnosis and treatment categories). If you filled out the behavioural & social sciences study design questions and have nothing to add here, write "See above."*
Recruitment	*Describe how participants were recruited. Outline any potential self-selection bias or other biases that may be present and how these are likely to impact results.*
Ethics oversight	*Identify the organization(s) that approved the study protocol.*

Note that full information on the approval of the study protocol must also be provided in the manuscript.

Field-specific reporting

Please select the one below that is the best fit for your research. If you are not sure, read the appropriate sections before making your selection.

☒ Life sciences ☐ Behavioural & social sciences ☐ Ecological, evolutionary & environmental sciences

For a reference copy of the document with all sections, see nature.com/documents/nr-reporting-summary-flat.pdf

Life sciences study design

All studies must disclose on these points even when the disclosure is negative.

Sample size	Experimental procedures involving mice followed the institutional guidelines for the care and use of laboratory animals and the ARRIVE guidelines. Protocols were approved by the Ethical Committee for Animal Experimentation of the University of Barcelona (CEEA-UB/Ref. 186/20-P2) and the Government of Catalonia (PAMN/Ref. 11485), which followed Directive 2010/63/EU for the protection of animals used for scientific purposes. The sample size was estimated by the Appraising Project Office's program from the Universitat Miguel Hernández of Elx (Alacant, Spain). We used 5-week-old male and female C57BL/6 mice (n = 16) purchased from Janvier and housed at the animal facility of the Faculty of Pharmacy and Food Science of the University of Barcelona in a controlled environment.
Data exclusions	No data were excluded.
Replication	The experiment and the control contained 8 animals each (4 male and 4 female mice), i.e. there were 8 replicates, and all attempts were successful.
Randomization	For each group, experiment and control, 4 male and 4 female mice were randomly selected.
Blinding	Researchers in charge were not blinded, because they had to specifically choose particular animals.

Reporting for specific materials, systems and methods

We require information from authors about some types of materials, experimental systems and methods used in many studies. Here, indicate whether each material, system or method listed is relevant to your study. If you are not sure if a list item applies to your research, read the appropriate section before selecting a response.

March 2021

Materials & experimental systems

n/a	Involved in the study
☐	☒ Antibodies
☐	☒ Eukaryotic cell lines
☒	☐ Palaeontology and archaeology
☐	☒ Animals and other organisms
☒	☐ Clinical data
☒	☐ Dual use research of concern

Methods

n/a	Involved in the study
☒	☐ ChIP-seq
☒	☐ Flow cytometry
☒	☐ MRI-based neuroimaging

Antibodies

Antibodies used	AgraQuant Gluten G12 ELISA test kit (Romer Labs)
Validation	The antibodies were employed directly from the purchased kit. We assumed that they had been checked by the manufacturer.

Eukaryotic cell lines

Policy information about cell lines and Sex and Gender in Research

Cell line source(s)	human Expi293F from Thermo Fisher Scientific
Authentication	Cells were not authenticated
Mycoplasma contamination	Cells were not tested for mycoplasma contamination
Commonly misidentified lines (See ICLAC register)	No commonly misidentified lines were used

Animals and other research organisms

Policy information about studies involving animals; ARRIVE guidelines recommended for reporting animal research, and Sex and Gender in Research

Laboratory animals	Five-week-old male and female C57BL/6 mice (n = 16) purchased from Janvier
Wild animals	No wild animals were used in this study
Reporting on sex	Sex was considered in the study and 8 female and 8 male mice were randomly assigned to the experiment and to control.
Field-collected samples	No field-collected samples were used in this study.
Ethics oversight	Experimental procedures involving mice followed the institutional guidelines for the care and use of laboratory animals and the ARRIVE guidelines. Protocols were approved by the Ethical Committee for Animal Experimentation of the University of Barcelona (CEEA-UB/Ref. 186/20-P2) and the Government of Catalonia (PAMN/Ref. 11485), which followed Directive 2010/63/EU for the protection of animals used for scientific purposes. The sample size was estimated by the Appraising Project Office's program from the Universitat Miguel Hernández of Elx (Alacant, Spain).

Note that full information on the approval of the study protocol must also be provided in the manuscript.
