# Supplementary material for: Molecular and in vivo studies of a glutamate-class prolyl-endopeptidase for coeliac disease therapy
Source: Nat Commun. 2022 Aug 1;13:4446. doi: 10.1038/s41467-022-32215-1 (PMC9343461; doi:10.1038/s41467-022-32215-1)

Original gels

Molecular and *in vivo* studies of a glutamate-class prolyl-endopeptidase for coeliac disease therapy

Laura del Amo-Maestro, Soraia R. Mendes, Arturo Rodríguez-Banqueri, Laura Garzon-Flores, Marina Girbal, María José Rodríguez-Lagunas, Tibisay Guevara, Àngels Franch, Francisco J. Pérez-Cano, Ulrich Eckhard and F. Xavier Gomis-Rüth

1A

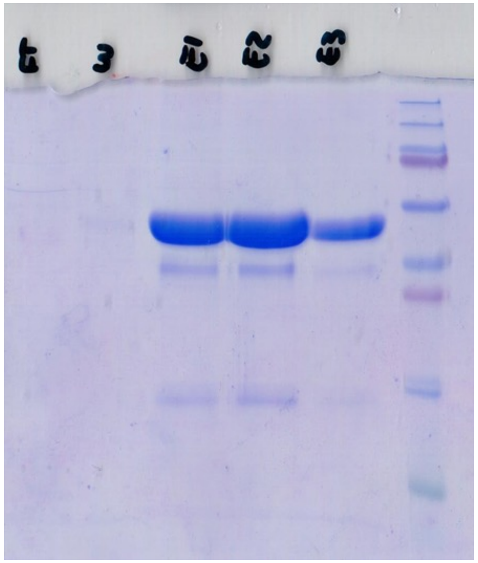

1B

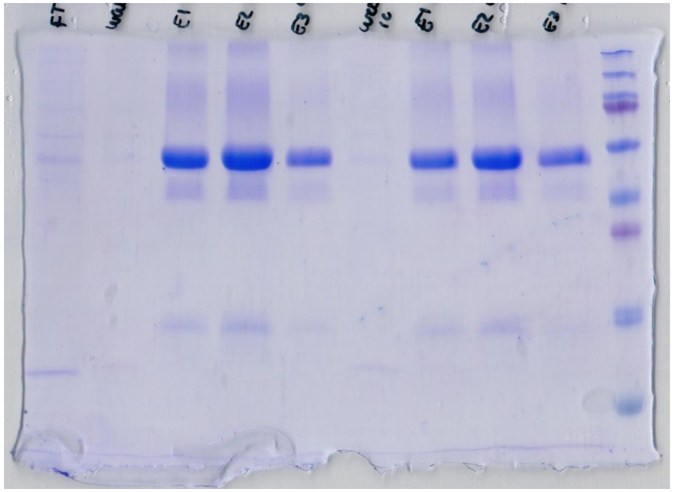

1C

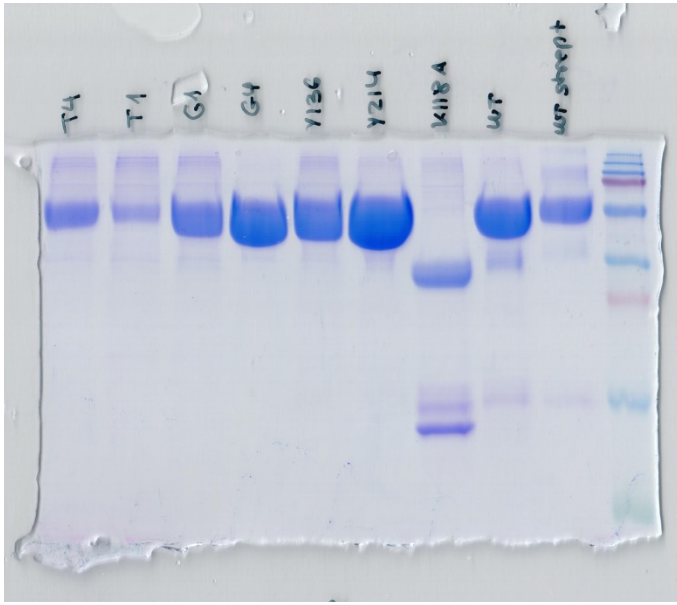

1C

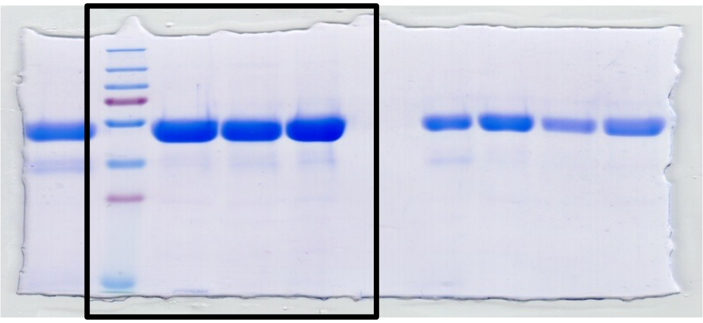

1F

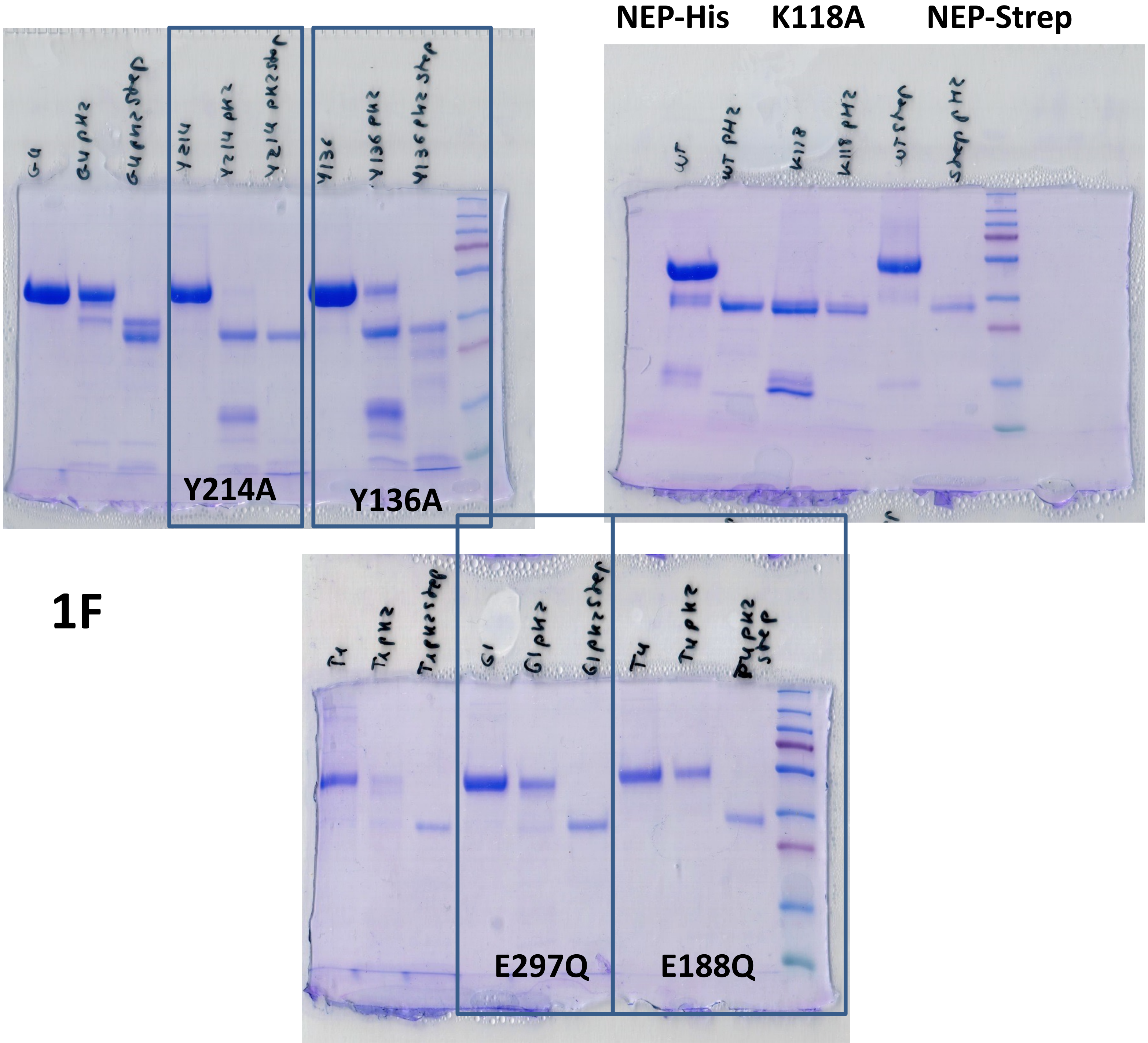

1E

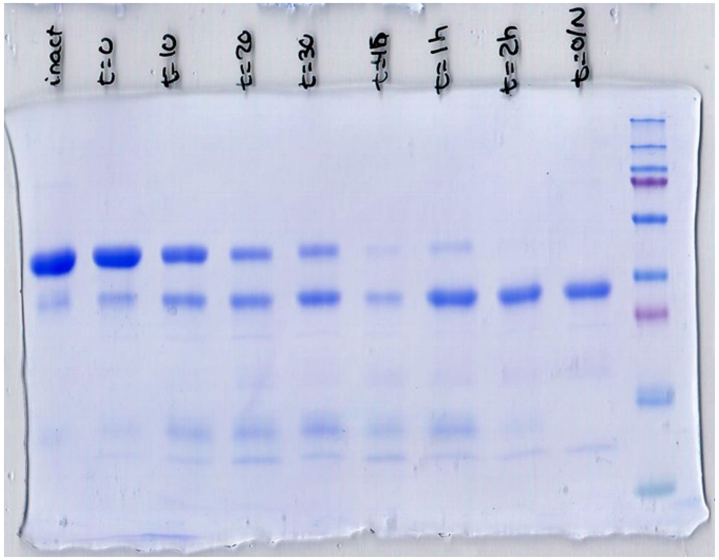

1F

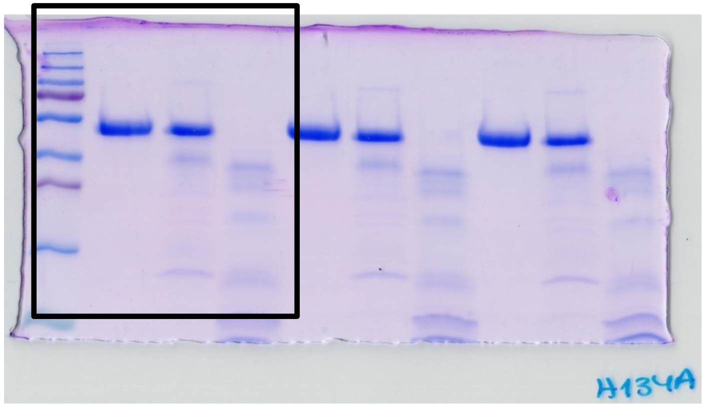

1F

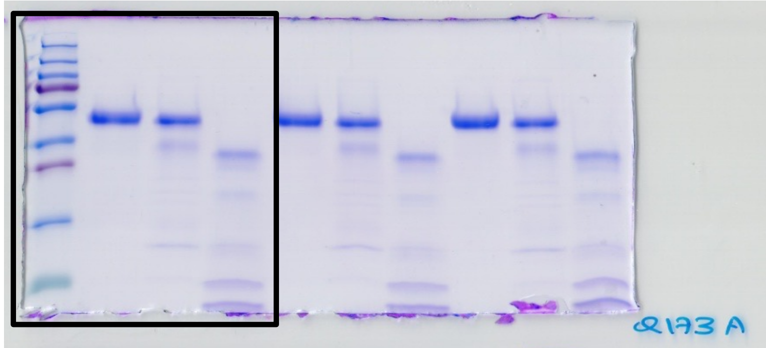

1F

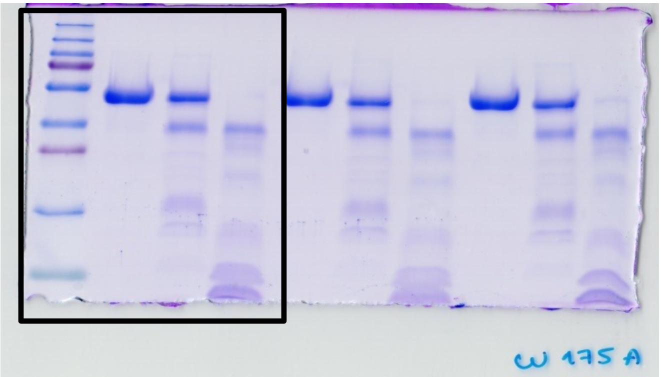

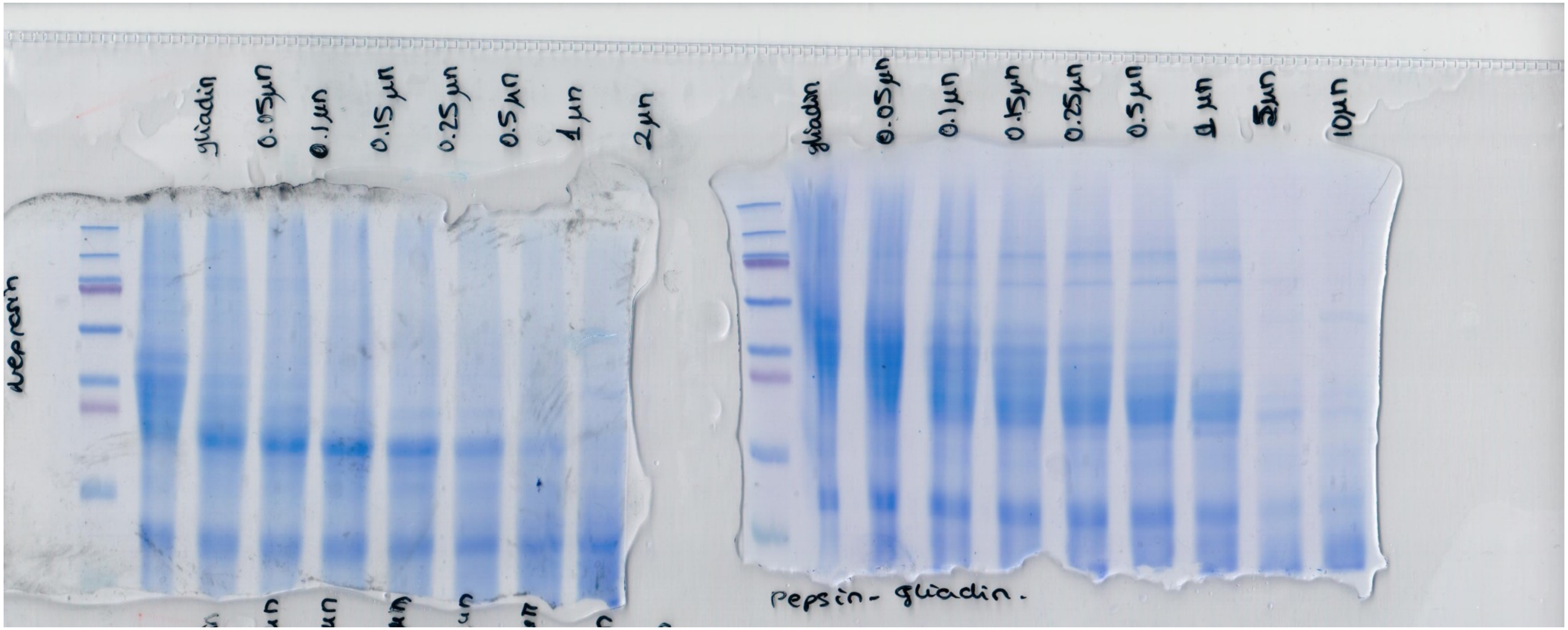
gliadin
0.05μn
0.1μn
0.15μn
0.25μn
0.5μn
1μn
2μn
Neprosin
gliadin
0.05μn
0.1μn
0.15μn
0.25μn
0.5μn
1μn
5μn
10μn
Pepsin- gliadin.

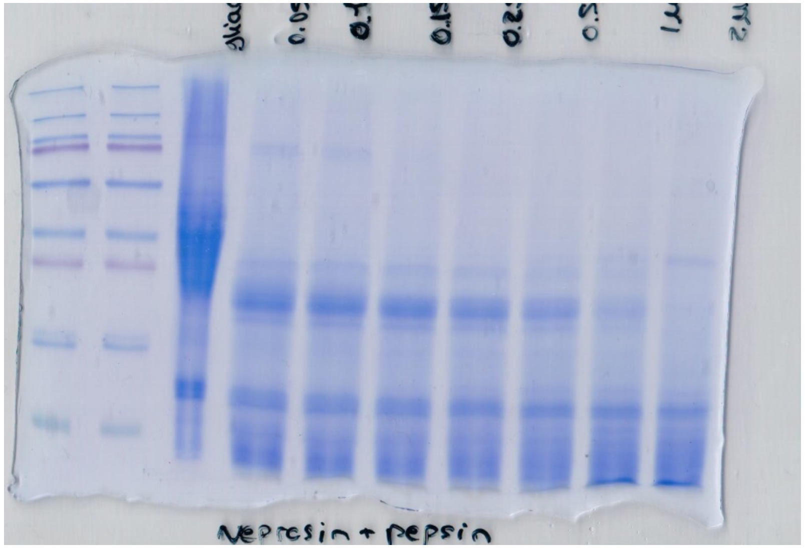
Neprosin + pepsin

3A

Figure 3D – Gliadin Zymogram

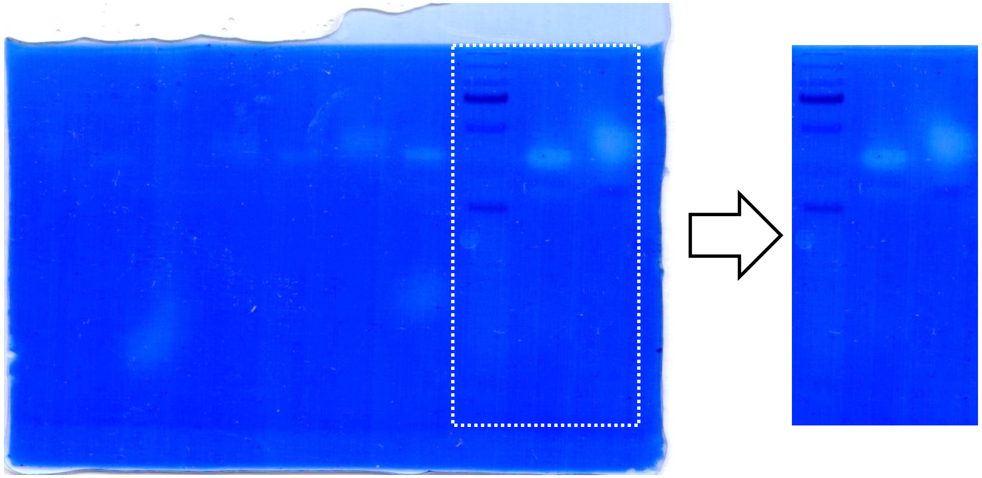

Figure 3E – Gelatin Zymogram

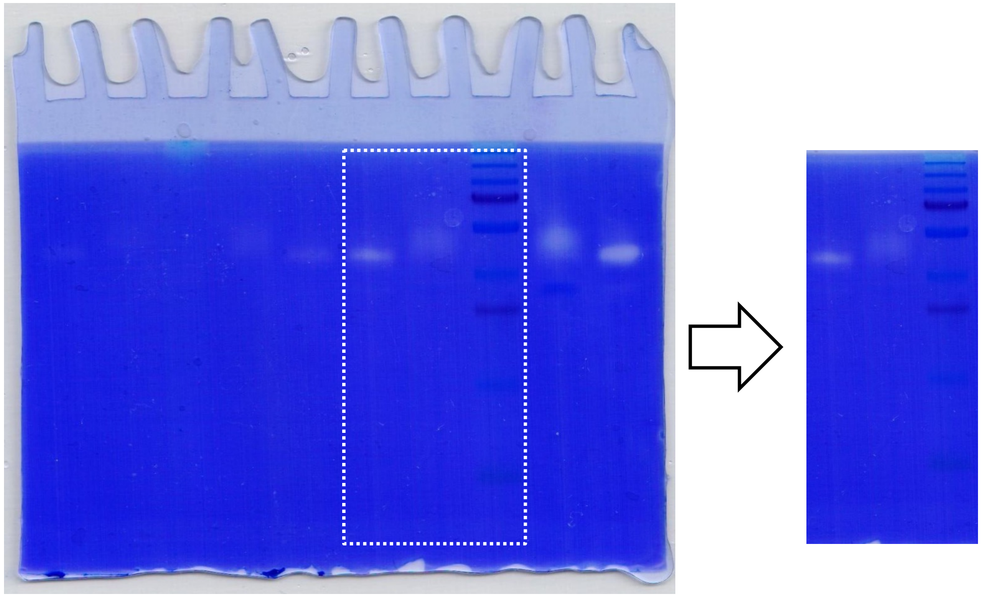

Supplement: Supplementary file 3 — Source Data [file 41467_2022_32215_MOESM3_ESM.zip › Original_gels.pdf]
